# Convenient Synthesis and Physiological Activities of Flavonoids in *Coreopsis lanceolata* L. Petals and Their Related Compounds

**DOI:** 10.3390/molecules23071671

**Published:** 2018-07-09

**Authors:** Daisuke Nakabo, Yuka Okano, Naomi Kandori, Taisei Satahira, Naoya Kataoka, Junpei Akamatsu, Yoshiharu Okada

**Affiliations:** Department of Biotechnology and Chemistry, Faculty of Engineering, Kindai University, Umenobe-1, Takaya, Higashi-hiroshima 739-2116 Japan; 1633850001b@hiro.kindai.ac.jp (D.N.); 1333850001@hiro.kindai.ac.jp (Y.O.); 1433850002@hiro.kindai.ac.jp (N.K.); 0910910069@hiro.kindai.ac.jp (T.S.); 1110910080@hiro.kindai.ac.jp (N.K.); 1410980093@hiro.kindai.ac.jp (J.A.)

**Keywords:** *Coreopsis lanceolata* L., chalcone, flavanone, flavonol, aurone, Horner–Wadsworth–Emmons reaction

## Abstract

Chalcones, flavanones, and flavonols, including 8-methoxybutin isolated from *Coreopsis lanceolata* L. petals, were successfully synthesized with total yields of 2–59% from *O*-methylpyrogallols using the Horner–Wadsworth–Emmons reaction as a key reaction. Aurones, including leptosidin, were also successfully synthesized with 5–36% total yields using the Aldol condensation reaction as a key reaction. Each chalcone, flavanone, flavonol, and aurone with the 3,4-dihydroxy groups in the B-ring showed high antioxidant activity. Additionally, each of the chalcones, flavanones, flavonols, and aurones with the 2,4-dihydroxy groups in the B-ring showed an excellent whitening ability.

## 1. Introduction

**Figure 1:**
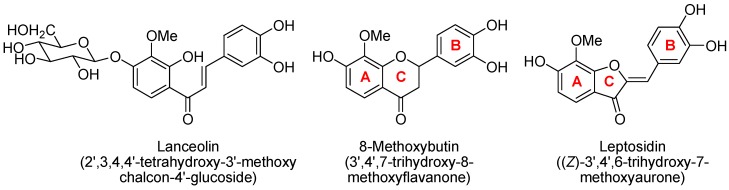
Isolated flavonoids from *Coreopsis lenceolata* L. petals.

*Coreopsis lanceolata* L. is a plant native to North America with a yellow flower that blooms from May to June in Japan. We previously reported the isolation and physiological activities of lanceolin (3,4,2′,4′-tetrahydroxy-3′-methoxychalcone-4′-glucoside), 8-methoxybutin (7,3′,4′-trihydroxy-8-methoxyflavanone), and leptosidin (6,3′,4′-trihydroxy-7-methoxyaurone) from *C. lanceolata* L. petals as shown in Figure 1 [1,2]. Koketsu et al. reported the isolation of lanceoletin (3,4,2′,4′-tetrahydroxy-3′-methoxychalcone), okanin (3,4,2′,3′,4′-pentahydroxychalcone), 4-methoxylanceoletin (3,2′,4′-trihydroxy-4,3′-dimethoxychalcone), 8-methoxybutin, leptosidin, and leptosin (6,3′,4′-trihydroxy-7-methoxyaurone-6-glucoside) from *C. lanceolata* L., and the antileukemic activity of 4-methoxylanceoletin [3]. In this paper, we report the synthesis of the several kinds of flavonoids including *C. lanceolata* L. petals and their analogs, and the relationship between structure and physiological activities.

## 2. Results and Discussion

### 2.1. Flavonoids Synthesis

**Scheme 1:**
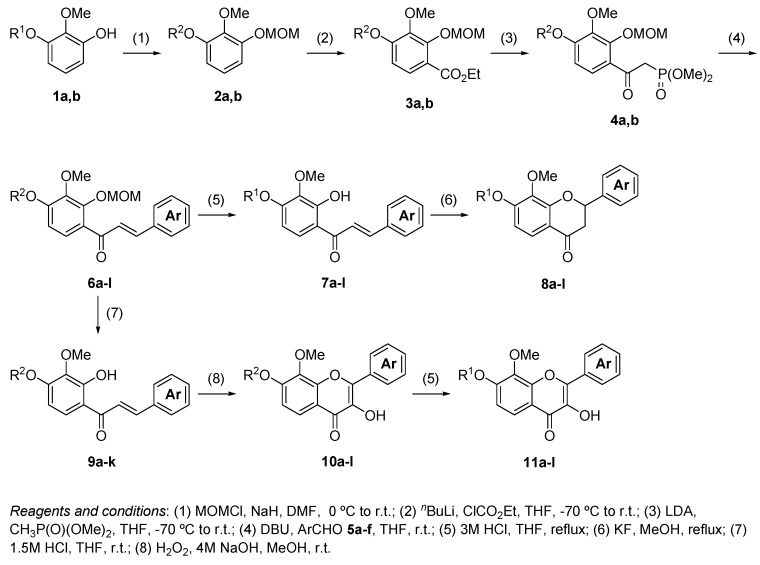
Synthesis of chalcones **7a–l**, flavanones **8a–l**, and flavonols **11a–l**.

The process used to synthesize the chalcones, flavanones, and flavonols is shown in Scheme 1. The protection of **1a**,**b** with chloromethyl methyl ether (MOMCl) produced compound **2a**,**b**. The lithiation at the 4-position of **2a**,**b** with *n*-BuLi, which was stabilized by the methoxymethoxy moiety and subsequent ethoxycarbonylation with ethyl chloroformate produced ethyl benzoates **3a**,**b** with 86 and 73% yields, respectively. The reaction of **3a**,**b** with dimethyl methylphosphonate in the presence of lithium diisopropylamide (LDA) produced β-keto phosphonates **4a**,**b** with 87 and 76% yields, respectively. The Horner–Wadsworth–Emmons (HWE) reaction, which is a key reaction in this process, of **4a**,**b** with aromatic aldehydes **5a**–**f** in the presence of 1,8-diazabicyclo[5.4.0]undec-7-ene (DBU) as a base produced the corresponding chalcone **6a**–**l** with 78–92% yields. Subsequently, the deprotection of **6a**–**l** with 3 M HCl at reflux produced the MOM group deprotected chalcones **7a**–**l** with 41–98% yields. The structures of **7a**–**l** were assigned based on their hydrogen and carbon nuclear magnetic resonance (^1^H-NMR and ^13^C-NMR) spectral data. The olefinic protons of **7a** were observed at δ 7.43 (d, *J* = 15.9 Hz) and δ 7.83 (d, *J* = 15.4 Hz), respectively. Therefore, the geometry of the double bond of **7a**–**l** was assigned as the (*E*)-form. A solution of **7a**–**l** in methanol containing potassium fluoride was heated at reflux to produce flavanones **8a**–**l** with 46–98% yields [4]. The ^1^H-NMR spectrum of **8a** shows a signal for the methine proton (dd, *J* = 2.9 and 12.9 Hz) at δ 5.42 and two methylene protons (dd, *J* = 3.2 and 17.1 Hz) at δ 2.72 and (dd, *J* = 12.9 and 16.9 Hz) at δ 3.07, respectively. These coupling constants of 2.9 Hz and 12.9 Hz were the vicinal coupling constants assigned to two methylene protons and a methine proton, respectively. Therefore, the structures of **8a**–**l** were determined as flavanones. Treatment of **6a**–**k** with 1.5 M HCl at room temperature produced the chalcones **9a**–**k** with 65–95% yields, which the 2′-MOM groups activated by the close carbonyl groups, which were selectively deprotected. Treatment of **9a**–**k** and **7l** with basic H_2_O_2_ produced the corresponding flavonols **10a**–**l** with 17–65% yields [5]. ^13^C-NMR spectrum of **10a** showed a signal for carbonyl carbon at δ 172.70. However, a similar signal for flavanone **8a** was observed at δ 193.21. The carbonyl carbon of **10a** shifted toward the upper field due to the influence of the double bond of flavonol. Therefore, the structures of **10a**–**l** were determined to be flavonols. Finally, the deprotection of the MOM groups of **10a**–**l** with 3 M HCl produced compounds **11a**–**l** with 38–98% yields.

**Scheme 2:**
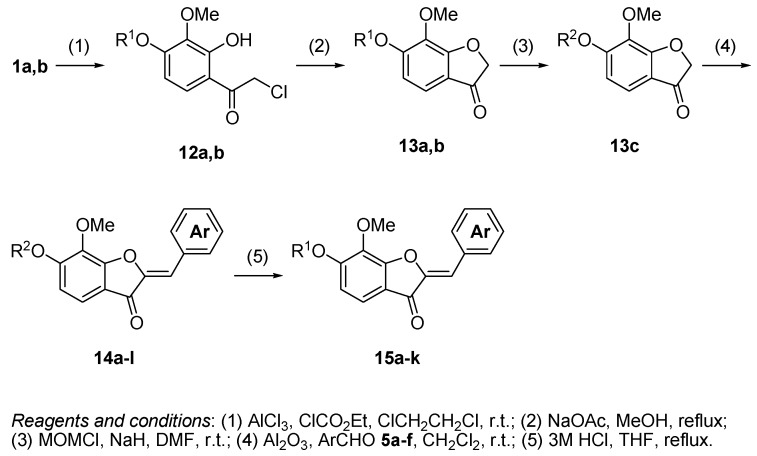
Synthesis of aurones **15a–k**.

The process used to synthesize the aurones is shown in Scheme 2. The Friedel–Crafts acylation of **1a**,**b** with chloroacetyl chloride produced compounds **12a**,**b** with 54 and 58% yields, respectively. The cyclization of **12b** with potassium hydroxide as a base produced benzofuranone **13b** with a low yield of 28% in a complex mixture since the intermolecular reaction of **12b** due to high basicity. Using sodium acetate instead of potassium hydroxide as a base resulted in an increase in the yield to 83%. A similar reaction of **12a** produced **13a** with a 77% yield. The protection of the hydroxyl group of **13a** with MOMCl produced compound **13c** with a 58% yield. The aldol condensation reaction, which is a key reaction in this process, of **13b**,**c** with aromatic aldehydes **5a**–**f** in the presence of aluminum oxide produced the corresponding aurones **14a**–**l** with 31–89% yields [6]. In this reaction, the use of **13a** resulted in decreasing yields. The structures of **14a**–**l** were classified on the basis of their ^1^H-NMR and ^13^C-NMR spectral data. The ^1^H-NMR spectrum of **14a** showed a signal for an olefinic proton at δ 6.84 (s). The olefinic carbon was observed at δ 112.16. According to the ^13^C-NMR study of the aurones, a signal for the olefinic carbon of the *Z*-isomer was observed at about 110 ppm, whereas that of *E*-isomer was observed at about 120 ppm [7]. Therefore, the structures of **14a**–**l** were classified as the (*Z*)-form. Finally, the deprotection of the MOM group of **14a**–**k** with 3 M HCl produced compounds **15a**–**k** with 51–97% yields.

### 2.2. Antioxidant and Tyrosinase Inhibitory Activity of the Synthesized Flavonoids

Next, the physiological activities of these synthesized compounds were investigated. The antioxidant activity and whitening effect were assessed based on the 2,2-diphenyl-1-picrylhydrazyl (DPPH) free radical scavenging assay [8] and tyrosinase inhibition assay [9], respectively. The results are summarized in Table 1, Table 2, Table 3 and Table 4. The antioxidant activity was evaluated based on the scavenging rate of the DPPH radical under the condition where the final concentration of the samples and DPPH radical were prepared at 0.040 mM and 0.040 mM, respectively. A correlation was found between the physiological activity and structures of the A-ring and B-ring of the chalcones, flavanones, flavonols, and aurones. On the chalcones, the hydroxyl group at the 4-position on the A-ring was confirmed to be important for the antioxidant activity since the chalcones **7g**–**l** with a methoxy group instead of a hydroxyl group at the 4-position displayed decreased activity. The flavanones **8a**–**f** showed lower antioxidant activity than chalcones **7a**–**f**, since the hydroxyl group at 2-position of the A-ring of chalcone was lost during the conversion into flavanone. For the aurones, **15g**–**k** with a methoxy substituent at the 6-position on the A-ring showed similar behavior. However, on the flavonols, the compounds **10l** and **11g**–**k** with methoxy groups at the 7-position on the A-ring showed a higher antioxidant activity than with hydroxyl groups. Each of the chalcones **7b**,**h**, flavanones **8b**,**h**, flavonols **11b**,**h**, and aurones **15b**,**h** with 3,4-dihydroxy groups on the B-ring had high antioxidant activity. In addition, each with a 4-hydroxy-3-methoxy placement on the B-ring (**7d**,**j**, **8d**,**j**, **11d**,**j**, and **15d**,**j**) had superior antioxidant effects compared with those with the 3-hydroxy-4-methoxy placement (**7e**,**k**, **8e**,**k**, **11e**,**k**, and **15e**,**k**). Moreover, the 4-hydroxyl group on the B-ring (**7j**, **8j**, **11j**, and **15j**) seemed to have more influence on the antioxidant activity than a hydroxyl group on the A-ring (**7f**, **8f**, **11f**, and **15f**). The flavanones and aurones showed low antioxidant activity; the correlation between the substitution groups and activity was recorded.

Although each of the chalcones **7c**,**i** and aurones **15c**,**i** with the 2,4-dihydroxy groups on the B-ring showed lower antioxidant activity, flavonols **11c**,**i** with those groups had a high activity due to an apparent enhancement by the hydroxyl group at the 3-position on the C-ring. Since flavanones showed lower radical scavenging activity overall, the double bond in the structure of the flavonoid was thought to strongly influence antioxidant activity. The antioxidant activity in decreasing order was flavonol, chalcone, aurone, and flavanone.

The whitening effect was evaluated by inhibition of tyrosinase activity under the condition where the final concentration of samples and tyrosinase were prepared in 0.10 mM and 20 units/mL, respectively. Each of chalcones **7b**,**h**, flavanones **8b**,**h**, flavonol **11h**, and aurones **15b**,**h** with 3,4-dihydroxy groups on the B-ring displayed a low inhibition rate. In addition, each with a 3-hydroxy-4-methoxy placement on the B-ring (**7e**,**k**, **8e**,**k**, **11e**,**k**, and **15e**,**k**) had a superior inhibition activity compared to those with the 4-hydroxy-3-methoxy placement (**7d**,**j**, **8d**,**j**, **11d**,**j**, and **15d**,**j**).

The chalcones **7c**,**i** and aurones **15c**,**i** bearing 2,4-dihydroxy groups on the B-ring demonstrated high inhibitory activity potential. Ramsden et al. explained that the reductive elimination and loss of copper atoms from the active site of tyrosinase via the resorcinol (1,3-dihydroxybenzene) moiety resulted in the inactivation of tyrosinase [10]. However, the flavonol **11c**,**i** bearing a similar group did not inhibit tyrosinase activity. This tendency could potentially be due to the steric hindrance of the hydroxyl group at the 3-position of the flavonol against the 2,4-dihydroxy groups on the B-ring. The whitening effect in decreasing order was chalcone, aurone. flavonol, and flavanone.

## 3. Materials and Methods

### 3.1. General Methods

^1^H-NMR and ^13^C-NMR spectra were obtained on a JEOL JNM-EX400 (Tokyo, Japan) spectrometer in CDCl_3_, CD_3_OD, or dimethyl sulfoxide (DMSO)-*d*_6_ operating at 400 MHz and 100 MHz, respectively, with Me_4_Si as the internal standard. The absorbance was measured with a microplate reader Corona MTP-300 (Tokyo, Japan). The absorbance was recorded in the 200–600 nm range at room temperature with Jasco V630 (Tokyo, Japan). The mass spectra were obtained on a Shimadzu gas chromatograph mass spectrometer (GCMS)-QP5000 (Kyoto, Japan) with a column temperature of 240 °C, injection temperature of 200 °C, and interface temperature of 230 °C, with He as the carrier gas at a flow rate of 1.3 mL/min. Tetrahydrofuran (THF) was purified by distillation over benzophenone ketyl under an argon atmosphere before use. The melting points were measured in open capillary tubes and were uncorrected.

### 3.2. The General Procedure for the Protection of 2-O-Methylpyrogallol ***1a,b*** with Chloromethyl Methyl Ether

A solution of **1a**,**b** (100.0 mmol) in *N*,*N*-dimethylformamide (DMF) (50 mL) was added to a suspension of sodium hydride (60% in mineral oil, 9.60 g, 240.0 mmol or 4.80 g, 120.0 mmol) in DMF (150 mL) at 0 °C. After being stirred at room temperature for 30 min, chloromethyl methyl ether (15.2 mL, 200.0 mmol or 11.4 mL, 150.0 mmol) was added to the mixture at 0 °C. After being stirred at room temperature for 6 h, 100 mL Et_2_O was added to the mixture. The reaction mixture was poured into ice water (400 mL). The mixture was extracted with Et_2_O. The organic layer was washed with water and brine and dried over anhydrous MgSO_4_. The solvent was evaporated in vacuo and the residue was chromatographed on silica gel with CHCl_3_-Et_2_O (9:1) to produce **2a**,**b**.

2-Methoxy-1,3-di(methoxymethoxy)benzene (**2a**): (22.14 g, 97.0 mmol, 97% yield); ^1^H-NMR (CDCl_3_) 3.52 (s, 6H, OCH_3_), 3.89 (s, 3H, OCH_3_), 5.22 (s, 4H, OCH_2_), 6.85 (d, *J* = 8.1 Hz, 2H, H-4 and H-6), 6.95 (t, *J* = 8.3 Hz, 1H, H-5).

1,2-Dimethoxy-3-(methoxymethoxy)benzene (**2b**): (18.63 g, 94.0 mmol, 94% yield); ^1^H-NMR (CDCl_3_) 3.52 (s, 3H, OCH_3_), 3.87 (s, 3H, OCH_3_), 3.87 (s, 3H, OCH_3_), 5.23 (s, 2H, OCH_2_), 6.63 (d, *J* = 8.1 Hz, 1H, H-6), 6.80 (d, *J* = 7.6 Hz, 1H, H-4), 6.98 (t, *J* = 8.3 Hz, 1H, H-5).

### 3.3. The General Procedure for the Preparation of Ethyl Benzoates ***3a,b***

*n*-BuLi (1.55 M hexane solution, 23.2 mL, 36.0 mmol) was added to a solution of **2a**,**b** (30.0 mmol) in THF (150 mL) at −70 °C. The reaction mixture was warmed to 0 °C and stirred for 90 min at the same temperature. The mixture was cooled to −70 °C and a solution of ethyl chloroformate (14.3 mL, 150.0 mmol) in THF (15 mL) was added dropwise to the mixture. After being stirred for 30 min at −70 °C, the mixture was stirred at room temperature for 2 h. The mixture was poured into an ice-saturated ammonium chloride aqueous solution. The mixture was extracted with Et_2_O. The organic layer was washed with water and brine and dried over anhydrous MgSO_4_. The solvent was evaporated in vacuo and the residue was chromatographed on silica gel with CHCl_3_-Et_2_O (9:1) to produce **3a**,**b**.

Ethyl 3-methoxy-2,4-di(methoxymethoxy)benzoate (**3a**): (7.75 g, 25.8 mmol, 86% yield); ^1^H-NMR (CDCl_3_) δ 1.38 (t, *J* = 7.1 Hz, 3H, CH_3_), 3.52 (s, 3H, OCH_3_), 3.62 (s, 3H, OCH_3_), 3.89 (s, 3H, OCH_3_), 4.37 (q, *J* = 7.1 Hz, 1H, OCH_2_CH_3_), 5.18 (s, 2H, OCH_2_), 5.28 (s, 2H, OCH_2_), 6.97 (d, *J* = 9.0 Hz, 1H, H-5), 7.60 (d, *J* = 8.8 Hz, 1H, H-6); ^13^C-NMR (CDCl_3_) δ 14.3, 56.4, 57.4, 60.8, 61.0, 94.6, 100.1, 110.6, 119.3, 126.7, 143.2, 151.3, 154.4, 165.1.

Ethyl 3,4-dimethoxy-2-(methoxymethoxy)benzoate (**3b**): (5.92 g, 21.9 mmol, 73% yield); ^1^H-NMR (CDCl_3_) δ 1.38 (t, *J* = 7.1 Hz, 3H, CH_3_), 3.61 (s, 3H, OCH_3_), 3.87 (s, 3H, OCH_3_), 3.91 (s, 3H, OCH_3_), 4.35 (q, *J* = 7.1 Hz, 1H, OCH_2_CH_3_), 5.17 (s, 2H, OCH_2_), 6.72 (d, *J* = 9.0 Hz, 1H, H-5), 7.65 (d, *J* = 8.8 Hz, 1H, H-6); ^13^C-NMR (CDCl_3_) δ 14.3, 56.0, 57.4, 60.7, 60.9, 100.0, 106.9, 118.2, 127.0, 142.5, 151.2, 156.8, 165.2.

### 3.4. The General Procedure for the Synthesis of α-(Dimethylphosphono)acetylbenzenes ***4a,b***

*n*-BuLi (1.55 M hexane solution, 42.6 mL, 66 mmol) was added to a solution of diisopropylamine (8.4 mL, 60.0 mmol) in THF (100 mL) at −70 °C. After being stirred for 30 min at the same temperature, a solution of dimethyl methylphosphonate (4.47 g, 36.0 mmol) in THF (15 mL) was added dropwise to the mixture. The reaction mixture was stirred for 15 min at −70 °C and a solution of **3a**,**b** (30.0 mmol) in THF (15 mL) was added to the mixture. The mixture was stirred for 1 h at −70 °C and for 12 h at room temperature. The mixture was poured into ice and a 2 M hydrochloric acid aqueous solution. The mixture was extracted with EtOAc. The organic layer was washed with water and brine and dried over anhydrous MgSO_4_. The solvent was evaporated in vacuo and the residue was chromatographed on silica gel with CHCl_3_-Et_2_O-MeOH (8:2:0.05) to produce **4a**,**b**.

α-(Dimethylphosphono)-3-methoxy-2,4-di(methoxymethoxy)acetophenone (**4a**): (9.87 g, 26.1 mmol, 87% yield); reddish brown viscous oil; ^1^H-NMR (CDCl_3_) δ 3.52 (s, 6H, OCH_3_), 3.77 (d, *J* = 11.2 Hz, 6H, P(O)OCH_3_), 3.86 (d, *J* = 21.5 Hz, 2H, P(O)CH_2_), 3.88 (s, 3H, OCH_3_), 5.23 (s, 2H, OCH_2_), 5.28 (s, 2H, OCH_2_), 6.98 (d, *J* = 8.8 Hz, 1H, H-5), 7.48 (d, *J* = 9.0 Hz, 1H, H-6); ^13^C-NMR (CDCl_3_) δ 40.3 (d, *J* = 131.0 Hz, P(O)CH_2_), 52.9 (d, *J* = 5.8 Hz, P(O)OCH_3_), 56.5, 58.0, 61.0, 94.7, 100.2, 110.9, 126.0, 126.9 (d, *J* = 3.3 Hz, Ar), 142.0, 150.6, 155.1, 191.9 (d, *J* = 6.6 Hz, CO).

α-(Dimethylphosphono)-3,4-dimethoxy-2-(methoxymethoxy)acetophenone (**4b**): (7.94 g, 22.8 mmol, 76% yield); reddish brown viscous oil; ^1^H-NMR (CDCl_3_) δ 3.51 (s, 3H, OCH_3_), 3.77 (d, *J* = 11.0 Hz, 6H, OCH_3_), 3.86 (s, 3H, OCH_3_), 3.87 (d, *J* = 21.7 Hz, 2H, P(O)CH_2_), 3.92 (s, 3H, OCH_3_), 5.23 (s, 2H, OCH_2_), 6.76 (d, *J* = 8.8 Hz, 1H, H-5), 7.54 (d, *J* = 8.8 Hz, 1H, H-6); ^13^C-NMR (CDCl_3_) δ 40.9 (d, *J* = 131.0 Hz, P(O)CH_2_), 52.8 (d, *J* = 6.6 Hz, P(O)OCH_3_), 56.1, 58.0, 60.8, 100.2, 107.3, 125.9 (d, *J* = 3.3 Hz, Ar), 126.2, 141.4, 150.6, 157.5, 191.7 (d, *J* = 6.6 Hz, CO).

### 3.5. The General Procedure for the Synthesis of Chalcones ***6a–l***

A solution of benzaldehydes **5a**–**f** (1.2 mmol) in THF (1 mL) was added to a solution of **4a**,**b** (1.0 mmol) and DBU (0.30 g, 2.0 mmol) in THF (4 mL) at room temperature. The reaction mixture was poured into an ice-saturated ammonium chloride aqueous solution and extracted with Et_2_O. The organic layer was washed with water and brine and dried over anhydrous MgSO_4_. The solvent was evaporated in vacuo and the residue was chromatographed on a preparative thin layer chromatography (hexane:EtOAc = 3:2) to produce chalcones **6a**–**l**.

3′-Methoxy-4,2′,4′-tri(methoxymethoxy)chalcone (**6a**): (0.35 g, 0.78 mmol, 78% yield); yellow viscous oil; ^1^H-NMR (CDCl_3_) δ 3.45 (s, 3H, OCH_3_), 3.49 (s, 3H, OCH_3_), 3.53 (s, 3H, OCH_3_), 3.93 (s, 3H, OCH_3_), 5.15 (s, 2H, OCH_2_), 5.21 (s, 2H, OCH_2_), 5.28 (s, 2H, OCH_2_), 7.00 (d, *J* = 8.8 Hz, 1H, H-5′), 7.06 (d, *J* = 8.5 Hz, 2H, H-3 and H-5), 7.34 (d, *J* = 15.9 Hz, 1H, H-α), 7.37 (d, *J* = 8.8 Hz, 1H, H-6′), 7.57 (d, *J* = 8.8 Hz, 2H, H-2 and H-6), 7.64 (d, *J* = 15.6 Hz, 1H, H-β); ^13^C-NMR (CDCl_3_) δ 56.0, 56.3, 57.5, 60.9, 94.0, 94.7, 99.9, 111.1, 116.2, 124.7, 125.0, 128.5, 128.8, 129.8, 142.4, 142.9, 149.8, 153.7, 158.7, 190.9.

3′-Methoxy-3,4,2′,4′-tetra(methoxymethoxy)chalcone (**6b**): (0.44 g, 0.91 mmol, 91% yield); yellow viscous oil; ^1^H-NMR (CDCl_3_) δ 3.46 (s, 3H, OCH_3_), 3.52 (s, 3H, OCH_3_), 3.54 (s, 6H, OCH_3_), 3.93 (s, 3H, OCH_3_), 5.16 (s, 2H, OCH_2_), 5.27 (s, 2H, OCH_2_), 5.28 (s, 2H, OCH_2_), 5.28 (s, 2H, OCH_2_), 7.00 (d, *J* = 8.8 Hz, 1H, H-5′), 7.17 (d, *J* = 8.5 Hz, 1H, H-5), 7.26 (dd, *J* = 2.0 and 8.3 Hz, 1H, H-6), 7.31 (d, *J* = 15.9 Hz, 1H, H-α), 7.36 (d, *J* = 8.8 Hz, 1H, H-6′), 7.43 (d, *J* = 2.0 Hz, H-2), 7.58 (d, *J* = 15.9 Hz, 1H, H-β); ^13^C-NMR (CDCl_3_) δ 56.1, 56.2, 56.3, 57.5, 60.9, 94.7, 94.9, 95.3, 99.8, 111.1, 116.0, 116.0, 123.5, 124.9, 125.2, 128.6, 129.2, 142.4, 143.1, 147.0, 149.0, 149.7, 153.7, 190.9.

3′-Methoxy-2,4,2′,4′-tetra(methoxymethoxy)chalcone (**6c**): (0.43 g, 0.89 mmol, 89% yield); yellow viscous oil; ^1^H-NMR (CDCl_3_) δ 3.47 (s, 3H, OCH_3_), 3.49 (s, 3H, OCH_3_), 3.50 (s, 3H, OCH_3_), 3.53 (s, 3H, OCH_3_), 3.93 (s, 3H, OCH_3_), 5.14 (s, 2H, OCH_2_), 5.20 (s, 2H, OCH_2_), 5.25 (s, 2H, OCH_2_), 5.29 (s, 2H, OCH_2_), 6.73 (dd, *J* = 2.2 Hz and 8.5 Hz, 1H, H-5), 6.84 (d, *J* = 2.2 Hz, 1H, H-3), 7.00 (d, *J* = 8.8 Hz, 1H, H-5′), 7.38 (d, *J* = 8.3 Hz, 1H, H-6′), 7.41 (d, *J* = 14.6 Hz, 1H, H-α), 7.60 (d, *J* = 8.5 Hz, 1H, H-6), 8.02 (d, *J* = 15.9 Hz, 1H, H-β); ^13^C-NMR (CDCl_3_) δ 56.3, 56.4, 56.5, 57.7, 61.1, 94.1, 94.5, 94.8, 100.0, 103.1, 109.2, 111.1, 118.4, 124.9, 125.2, 129.2, 129.3, 138.3, 142.5, 149.9, 153.7, 157.4, 160.0, 191.3.

3,3′-Dimethoxy-4,2′,4′-tri(methoxymethoxy)chalcone (**6d**): (0.37 g, 0.82 mmol, 82% yield); ^1^H-NMR (CDCl_3_) δ 3.46 (s, 3H, OCH_3_), 3.52 (s, 3H, OCH_3_), 3.54 (s, 3H, OCH_3_), 3.93 (s, 6H, OCH_3_), 5.16 (s, 2H, OCH_2_), 5.28 (s, 2H, OCH_2_), 5.29 (s, 2H, OCH_2_), 7.00 (d, *J* = 8.8 Hz, 1H, H-5′), 7.16–7.17 (m, 3H, H-2, H-5 and H-6), 7.36 (d, *J* = 15.9 Hz, 1H, H-α), 7.37 (d, *J* = 8.5 Hz, 1H, H-6′), 7.61 (d, *J* = 15.9 Hz, 1H, H-β); ^13^C-NMR (CDCl_3_) δ 55.8, 56.2, 56.3, 57.5, 60.9, 94.7, 95.0, 99.8, 110.6, 111.1, 115.6, 122.3, 124.9, 125.0, 128.6, 129.1, 142.4, 143.2, 148.3, 149.5, 149.7, 153.7, 190.9.

4,3′-Dimethoxy-3,2′,4′-tri(methoxymethoxy)chalcone (**6e**): (0.36 g, 0.80 mmol, 80% yield); ^1^H-NMR (CDCl_3_) δ 3.46 (s, 3H, OCH_3_), 3.53 (s, 6H, OCH_3_), 3.92 (s, 3H, OCH_3_), 3.93 (s, 3H, OCH_3_), 5.16 (s, 2H, OCH_2_), 5.26 (s, 2H, OCH_2_), 5.28 (s, 2H, OCH_2_), 6.91 (d, *J* = 8.3 Hz, 1H, H-5), 7.00 (d, *J* = 8.8 Hz, 1H, H-5′), 7.26 (dd, *J* = 1.7 Hz and 8.3 Hz, 1H, H-6), 7.29 (d, *J* = 15.6 Hz, 1H, H-α), 7.35 (d, *J* = 8.8 Hz, 1H, H-6′), 7.43 (d, *J* = 1.7 Hz, 1H, H-2), 7.59 (d, *J* = 15.6 Hz, 1H, H-β); ^13^C-NMR (CDCl_3_) δ 55.9, 56.3, 56.4, 57.6, 61.0, 94.9, 95.5, 99.9, 111.2, 111.5, 115.6, 124.1, 124.9, 125.1, 128.0, 128.9, 142.6, 143.5, 146.6, 149.9, 151.7, 153.8, 191.3.

3,4,3′-Trimethoxy-2′,4′-di(methoxymethoxy)chalcone (**6f**): (0.35 g, 0.83 mmol, 83% yield); ^1^H-NMR (CDCl_3_) δ 3.47 (s, 3H, OCH_3_), 3.54 (s, 3H, OCH_3_), 3.93 (s, 3H, OCH_3_), 3.93 (s, 6H, OCH_3_), 5.17 (s, 2H, OCH_2_), 5.29 (s, 2H, OCH_2_), 6.88 (d, *J* = 8.3 Hz, 1H, H-5), 7.00 (d, *J* = 8.8 Hz, H-5′), 7.15 (d, *J* = 2.0 Hz, 1H, H-2), 7.20 (dd, *J* = 2.0 Hz and 8.3 Hz, 1H, H-6), 7.32 (d, *J* = 15.9 Hz, 1H, H-α), 7.37 (d, *J* = 8.5 Hz, 1H, H-6′), 7.62 (d, *J* = 15.9 Hz, 1H, H-β); ^13^C-NMR (CDCl_3_) δ 55.9, 56.0, 56.5, 57.7, 61.1, 94.9, 100.0, 110.0, 111.0, 111.3, 123.1, 124.7, 125.2, 127.9, 128.9, 143.7, 149.1, 150.0, 151.2, 153.9, 191.2.

3′,4′-Dimethoxy-4,2′-di(methoxymethoxy)chalcone (**6g**): (0.35 g, 0.89 mmol, 89% yield); yellow viscous oil; ^1^H-NMR (CDCl_3_) δ 3.45 (s, 3H, OCH_3_), 3.49 (s, 3H, OCH_3_), 3.91 (s, 3H, OCH_3_), 3.93 (s, 3H, OCH_3_), 5.15 (s, 2H, OCH_2_), 5.22 (s, 2H, OCH_2_), 6.78 (d, *J* = 8.8 Hz, 1H, H-5′), 7.06 (d, *J* = 8.5 Hz, 2H, H-3 and H-5), 7.39 (d, *J* = 15.9 Hz, 1H, H-α), 7.45 (d, *J* = 8.5 Hz, 1H, H-6′), 7.58 (d, *J* = 8.8 Hz, 2H, H-2 and H-6), 7.66 (d, *J* = 15.9 Hz, 1H, H-β); ^13^C-NMR (CDCl_3_) δ 56.1, 56.2, 57.7, 61.0, 94.1, 100.1, 107.4, 116.3, 124.8, 125.5, 127.8, 128.7, 129.9, 141.7, 142.8, 150.0, 156.4, 158.8, 190.8.

3′,4′-Dimethoxy-3,4,2′-tri(methoxymethoxy)chalcone (**6h**): (0.35 g, 0.79 mmol, 79% yield); ^1^H-NMR (CDCl_3_) δ 3.46 (s, 3H, OCH_3_), 3.52 (s, 3H, OCH_3_), 3.54 (s, 3H, OCH_3_), 3.91 (s, 3H, OCH_3_), 3.93 (s, 3H, OCH_3_), 5.16 (s, 2H, OCH_2_), 5.28 (s, 2H, OCH_2_), 5.29 (s, 2H, OCH_2_), 6.78 (d, *J* = 8.8 Hz, 1H, H-5′), 7.18 (d, *J* = 8.5 Hz, 1H, H-5), 7.26 (dd, *J* = 2.0 Hz and 8.5 Hz, 1H, H-6), 7.36 (d, *J* = 15.9 Hz, 1H, H-α), 7.44 (d, *J* = 8.5 Hz, 1H, H-6′), 7.44 (d, *J* = 2.0 Hz, 1H, H-2), 7.60 (d, *J* = 15.6 Hz, 1H, H-β); ^13^C-NMR (CDCl_3_) δ 56.0, 56.2, 56.3, 57.6, 60.9, 94.9, 95.3, 99.9, 107.3, 116.0, 116.0, 123.6, 125.3, 125.4, 127.6, 129.3, 141.7, 143.0, 147.1, 149.0, 150.0, 156.4, 191.0.

3′,4′-Dimethoxy-2,4,2′-tri(methoxymethoxy)chalcone (**6i**): (0.37 g, 0.83 mmol, 83% yield); yellow viscous oil; ^1^H-NMR (CDCl_3_) δ 3.46 (s, 3H, OCH_3_), 3.49 (s, 3H, OCH_3_), 3.50 (s, 3H, OCH_3_), 3.91 (s, 3H, OCH_3_), 3.93 (s, 3H, OCH_3_), 5.14 (s, 2H, OCH_2_), 5.20 (s, 2H, OCH_2_), 5.25 (s, 2H, OCH_2_), 6.73 (dd, *J* = 2.2 and 8.5 Hz, 1H, H-5), 6.77 (d, *J* = 8.8 Hz, 1H, H-5′), 6.84 (d, *J* = 2.2 Hz, 1H, H-3), 7.45 (d, *J* = 15.9 Hz, 1H, H-α), 7.45 (d, *J* = 8.8 Hz, 1H, H-6′), 7.61 (d, *J* = 8.5 Hz, 1H, H-6), 8.03 (d, *J* = 15.9 Hz, 1H, H-β); ^13^C-NMR (CDCl_3_) δ 55.9, 56.1, 56.2, 57.5, 60.8, 94.0, 94.4, 99.8, 103.0, 107.2, 109.1, 118.4, 124.8, 125.3, 127.9, 129.1, 137.9, 141.6, 149.7, 156.1, 157.3, 159.8, 191.0.

3,3′,4′-Trimethoxy-4,2′-di(methoxymethoxy)chalcone (**6j**): (0.33 g, 0.78 mmol, 78% yield); yellow viscous oil; ^1^H-NMR (CDCl_3_) δ 3.46 (s, 3H, OCH_3_), 3.52 (s, 3H, OCH_3_), 3.91 (s, 3H, OCH_3_), 3.93 (s, 6H, OCH_3_), 5.17 (s, 2H, OCH_2_), 5.28 (s, 2H, OCH_2_), 6.78 (d, *J* = 8.8 Hz, 1H, H-5′), 7.17 (br s, 3H, H-2, H-5, and H-6), 7.39 (d, *J* = 15.6 Hz, 1H, H-α), 7.45 (d, *J* = 8.8 Hz, 1H, H-6′), 7.63 (d, *J* = 15.9 Hz, 1H, H-β); ^13^C-NMR (CDCl_3_) δ 55.9, 56.0, 56.3, 57.6, 60.9, 95.1, 100.0, 107.4, 110.7, 115.6, 122.5, 125.1, 125.5, 127.6, 129.3, 141.7, 143.1, 148.4, 149.6, 150.0, 156.5, 190.8.

4,3′,4′-Trimethoxy-3,2′-di(methoxymethoxy)chalcone (**6k**): (0.37 g, 0.89 mmol, 89% yield); yellow viscous oil; ^1^H-NMR (CDCl_3_) δ 3.46 (s, 3H, OCH_3_), 3.53 (s, 3H, OCH_3_), 3.91 (s, 3H, OCH_3_), 3.92 (s, 3H, OCH_3_), 3.93 (s, 3H, OCH_3_), 5.16 (s, 2H, OCH_2_), 5.26 (s, 2H, OCH_2_), 6.77 (d, *J* = 8.8 Hz, 1H, H-5′), 6.91 (d, *J* = 8.5 Hz, 1H, H-5), 7.27 (dd, *J* = 2.0 Hz and 8.3 Hz, 1H, H-6), 7.34 (d, *J* = 15.9 Hz, 1H, H-α), 7.43 (d, *J* = 8.5 Hz, 1H, H-6′), 7.44 (d, *J* = 1.7 Hz, 1H, H-2), 7.60 (d, *J* = 15.6 Hz, 1H, H-β); ^13^C-NMR (CDCl_3_) δ 55.9, 56.0, 56.2, 57.5, 60.8, 95.3, 99.8, 107.2, 111.4, 115.5, 123.9, 124.7, 125.2, 127.6, 127.9, 141.6, 143.0, 146.3, 149.7, 151.5, 156.2, 190.7.

3,4,3′,4′-Tetramethoxy-2′-(methoxymethoxy)chalcone (**6l**): (0.36 g, 0.92 mmol, 92% yield); ^1^H-NMR (CDCl_3_) δ 3.46 (s, 3H, OCH_3_), 3.91 (s, 3H, OCH_3_), 3.93 (s, 3H, OCH_3_), 3.93 (s, 3H, OCH_3_), 3.93 (s, 3H, OCH_3_), 5.17 (s, 2H, OCH_2_), 6.78 (d, *J* = 8.8 Hz, 1H, H-5′), 6.89 (d, *J* = 8.3 Hz, 1H, H-5), 7.16 (d, *J* = 2.0 Hz, 1H, H-2), 7.20 (dd, *J* = 2.0 Hz and 8.3 Hz, 1H, H-6), 7.37 (d, *J* = 15.9 Hz, 1H, H-α), 7.44 (d, *J* = 8.5 Hz, 1H, H-6′), 7.63 (d, *J* = 15.9 Hz, 1H, H-β); ^13^C-NMR (CDCl_3_) δ 55.8, 55.9, 56.0, 57.6, 60.9, 100.0, 107.4, 109.9, 110.9, 123.0, 124.6, 125.4, 127.7, 127.9, 141.7, 143.3, 149.0, 149.9, 151.0, 156.4, 190.9.

### 3.6. The General Procedure for the Deprotection of ***6a–l***

A solution of **6a**–**l** (1.0 mmol) in methanol (5 mL) and 3 M hydrochloric acid (5 mL) was refluxed for 1 h. The mixture was extracted with EtOAc. The organic layer was washed with water and brine and dried over anhydrous MgSO_4_. The solvent was evaporated in vacuo and the residue was chromatographed on preparative thin layer chromatography (hexane:EtOAc = 2:3) to produce aurones **7a**–**l**.

4,2′,4′-Trihydroxy-3′-methoxychalcone (**7a**): (0.22 g, 0.76 mmol, 76% yield); yellowish brown solid, 204–209 °C; ^1^H-NMR (CDCl_3_:DMSO-*d*_6_ = 9:1) δ 3.93 (s, 3H, OCH_3_), 6.53 (d, *J* = 8.8 Hz, 1H, H-5′), 6.90 (d, *J* = 7.8 Hz, 2H, H-3 and H-5), 7.43 (d, *J* = 15.9 Hz, 1H, H-α), 7.54 (d, *J* = 8.1 Hz, 2H, H-2 and H-6), 7.61 (d, *J* = 8.8 Hz, 1H, H-6′), 7.83 (d, *J* = 15.4 Hz, 1H, H-β), 9.25 (s, 1H, OH), 9.57 (s, 1H, OH), 13.76 (s, 1H, OH); ^13^C-NMR (CDCl_3_:DMSO-*d*_6_ = 9:1) δ 60.3, 107.4, 114.0, 115.9, 116.4, 125.7, 125.7, 130.2, 134.6, 144.3, 156.2, 158.3, 159.9, 191.7; UV/Vis (3.0 × 10^−5^ M, DMSO); λ = 380.0 nm (ε, 3.0 × 10^4^).

3,4,2′,4′-Tetrahydroxy-3′-methoxychalcone (**7b**): (0.25 g, 0.84 mmol, 84% yield); yellow solid, 191–195 °C; ^1^H-NMR (DMSO-*d*_6_) δ 3.74 (s, 3H, OCH_3_), 6.48 (d, *J* = 9.3 Hz, 1H, H-5′), 6.82 (d, *J* = 8.3 Hz, 1H, H-5), 7.22 (dd, *J* = 2.0 Hz and 8.3 Hz, 1H, H-6), 7.29 (d, *J* = 2.0 Hz, 1H, H-2), 7.65 (d, *J* = 15.1 Hz, 1H, H-α), 7.69 (d, *J* = 15.1 Hz, 1H, H-β), 7.93 (d, *J* = 9.3 Hz, 1H, H-6′), 9.12 (s, 1H, OH), 9.71 (s, 1H, OH), 10.40 (s, 1H, OH), 13.74 (s, 1H, OH); ^13^C-NMR (DMSO-*d*_6_) δ 59.7, 107.9, 113.7, 115.7, 115.9, 117.2, 122.4, 126.2, 126.8, 134.7, 144.9, 145.6, 149.0, 157.1, 158.5, 192.0; UV/Vis (2.6 × 10^−5^ M, DMSO); λ = 400.5 nm (ε, 2.1 × 10^4^).

2,4,2′,4′-Tetrahydroxy-3′-methoxychalcone (**7c**): (0.14 g, 0.47 mmol, 47% yield); reddish orange solid, 189–193 °C; ^1^H-NMR (CD_3_OD) δ 3.85 (s, 3H, OCH_3_), 6.34 (d, *J* = 2.2 Hz, 1H, H-3), 6.36 (dd, *J* = 2.2 Hz and 9.5 Hz, 1H, H-5), 6.46 (d, *J* = 8.8 Hz, 1H, H-5′), 7.51 (d, *J* = 8.3 Hz, 1H, H-6), 7.68 (d, *J* = 9.5 Hz, 1H, H-6′), 7.69 (d, *J* = 15.1 Hz, 1H, H-α), 8.10 (d, *J* = 15.4 Hz, 1H, H-β); ^13^C-NMR (CD3OD) δ 60.8, 103.4, 108.6, 109.0, 115.4, 115.5, 117.4, 127.2, 132.3, 136.0, 142.2, 157.6, 159.3, 160.6, 162.6, 194.3; UV/Vis (2.8 × 10^−5^ M, DMSO); λ = 399.0 nm (ε, 3.0 × 10^4^).

4,2′,4′-Trihydroxy-3,3′-dimethoxychalcone (**7d**): (0.30 g, 0.95 mmol, 95% yield); orange solid, 158–163 °C; ^1^H-NMR (DMSO-*d*_6_) δ 3.76 (s, 3H, OCH_3_), 3.89 (s, 3H, OCH_3_), 6.51 (d, *J* = 9.0 Hz, 1H, H-5′), 6.86 (d, *J* = 8.1 Hz, 1H, H-5), 7.30 (d, *J* = 8.3 Hz, 1H, H-6), 7.56 (br s, 1H, H-2), 7.76 (d, *J* = 15.1 Hz, 1H, H-α), 7.81 (d, *J* = 15.4 Hz, 1H, H-β), 8.01 (d, *J* = 8.8 Hz, 1H, H-6′), 9.77 (s, 1H, OH), 10.46 (s, 1H, OH), 13.83 (s, 1H, OH); ^13^C-NMR (DMSO-*d*_6_) δ 55.8, 59.7, 107.7, 111.5, 113.5, 115.4, 117.2, 124.4, 125.9, 126.7, 134.5, 144.6, 147.7, 149.7, 156.9, 158.2, 191.7; UV/Vis (2.5 × 10^−5^ M, DMSO); λ = 397.5 nm (ε, 2.8 × 10^4^).

3,2′,4′-Trihydroxy-4,3′-dimethoxychalcone (**7e**): (0.29 g, 0.90 mmol, 90% yield); yellowish brown solid, 180–185 °C; ^1^H-NMR (DMSO-*d*_6_) δ 3.77 (s, 3H, OCH_3_), 3.87 (s, 3H, OCH_3_), 6.52 (d, *J* = 8.8 Hz, 1H, H-5′), 7.02 (d, *J* = 8.5 Hz, 1H, H-5), 7.34 (dd, *J* = 1.7 Hz and 8.3 Hz, 1H, H-6), 7.39 (d, *J* = 1.5 Hz, 1H, H-2), 7.73 (d, *J* = 15.4 Hz, 1H, H-α), 7.78 (d, *J* = 15.4 Hz, 1H, H-β), 8.00 (d, *J* = 9.0 Hz, 1H, H-6), 9.24 (s, 1H, OH), 10.54 (s, 1H, OH), 13.76 (s, 1H, OH); ^13^C-NMR (DMSO-*d*_6_) δ 55.7, 59.8, 107.9, 111.7, 113.6, 114.9, 118.1, 122.2, 126.8, 127.3, 134.5, 144.3, 146.4, 150.2, 157.0, 158.3, 191.7; UV/Vis (2.7 × 10^−5^ M, DMSO); λ = 400.0 nm (ε, 2.2 × 10^4^).

2′,4′-Dihydroxy-3,4,3′-trimethoxychalcone (**7f**): (0.32 g, 0.98 mmol, 98% yield); orange-yellow solid, 140–144 °C; ^1^H-NMR (DMSO-*d*_6_) δ 3.75 (s, 3H, OCH_3_), 3.83 (s, 3H, OCH_3_), 3.88 (s, 3H, OCH_3_), 6.51 (d, *J* = 9.0 Hz, 1H, H-5′), 7.04 (d, *J* = 8.3 Hz, 1H, H-5), 7.57 (d, *J* = 2.0 Hz, 1H, H-2), 7.41 (dd, *J* = 2.0 Hz and 8.3 Hz, 1H, H-6), 7.78 (d, *J* = 15.4 Hz, 1H, H-α), 7.86 (d, *J* = 15.4 Hz, 1H, H-β), 8.02 (d, *J* = 9.0 Hz, 1H, H-6′), 10.48 (s, 1H, OH), 13.76 (s, 1H, OH); ^13^C-NMR (DMSO-*d*_6_) δ 55.6, 55.7, 59.7, 107.7, 110.6, 111.3, 113.5, 118.2, 124.2, 126.8, 127.1, 134.5, 144.2, 148.7, 151.2, 157.0, 158.2, 191.7; UV/Vis (3.3 × 10^−5^ M, DMSO); λ = 394.0 nm (ε, 2.3 × 10^4^).

4,2′-Dihydroxy-3′,4′-dimethoxychalcone (**7g**): (0.25 g, 0.84 mmol, 84% yield); yellow solid, 152–156 °C; ^1^H-NMR (CDCl_3_) δ 3.93 (s, 3H, OCH_3_), 3.96 (s, 3H, OCH_3_), 5.75 (s, 1H, OH), 6.54 (d, *J* = 9.0 Hz, 1H, H-5′), 6.90 (d, *J* = 8.5 Hz, 2H, H-3 and H-5), 7.45 (d, *J* = 15.4 Hz, 1H, H-α), 7.57 (d, *J* = 8.5 Hz, 2H, H-2 and H-6), 7.69 (d, *J* = 9.0 Hz, 1H, H-6′), 7.86 (d, *J* = 15.4 Hz, 1H, H-β), 13.35 (s, 1H, OH); ^13^C-NMR (CDCl_3_) δ 56.0, 60.6, 102.6, 115.4, 115.8, 117.4, 125.6, 127.2, 130.3, 136.3, 144.3, 157.8, 157.9, 158.0, 192.1.

3,4,2′-Trihydroxy-3′,4′-dimethoxychalcone (**7h**): (0.16 g, 0.51 mmol, 51% yield); green-yellow solid, 147–151 °C; ^1^H-NMR (CDCl_3_) δ 3.93 (s, 3H, OCH_3_), 3.95 (s, 3H, OCH_3_), 5.91 (s, 1H, OH), 6.01 (s, 1H, OH), 6.54 (d, *J* = 9.0 Hz, 1H, H-5′), 6.92 (d, *J* = 8.1 Hz, 1H, H-5), 7.14 (d, *J* = 8.3 Hz, 1H, H-6), 7.18 (s, 1H, H-2), 7.39 (d, *J* = 15.1 Hz, 1H, H-α), 7.67 (d, *J* = 9.0 Hz, 1H, H-6′), 7.77 (d, *J* = 15.4 Hz, 1H, H-β), 13.39 (s, 1H, OH); ^13^C-NMR (CD3OD) δ 56.5, 60.8, 104.2, 115.8, 116.5, 116.7, 117.9, 123.7, 127.6, 128.1, 137.3, 146.6, 146.7, 150.0, 158.5, 159.6, 193.9.

2,4,2′-Trihydroxy-3′,4′-methoxychalcone (**7i**): (0.13 g, 0.41 mmol, 41% yield); reddish orange solid, 120–125 °C; ^1^H-NMR (DMSO-*d*_6_) δ 3.69 (s, 3H, OCH_3_), 3.87 (s, 3H, OCH_3_), 6.33 (dd, *J* = 1.7 Hz and 8.8 Hz, 1H, H-5), 6.38 (d, *J* = 1.7 Hz, 1H, H-3), 6.68 (d, *J* = 8.8 Hz, 1H, H-5′), 7.70 (d, *J* = 14.9 Hz, 1H, H-α), 7.73 (d, *J* = 7.6 Hz, 1H, H-6′), 7.95 (d, *J* = 9.0 Hz, 1H, H-6), 8.09 (d, *J* = 15.4 Hz, 1H, H-β), 10.33 (br s, 2H, OH), 13.57 (s, 1H, OH); ^13^C-NMR (DMSO-*d*_6_) δ 56.2, 59.9, 102.4, 103.5, 108.2, 113.2, 115.2, 115.4, 126.5, 130.6, 135.7, 140.5, 157.1, 157.9, 159.3, 161.8, 192.4.

4,2′-Dihydroxy-3,3′,4′-trimethoxychalcone (**7j**): (0.25 g, 0.77 mmol, 77% yield); yellowish brown solid, 117–124 °C; ^1^H-NMR (CDCl_3_) δ 3.93 (s, 3H, OCH_3_), 3.96 (s, 3H, OCH_3_), 3.98 (s, 3H, OCH_3_), 5.96 (s, 1H, OH), 6.54 (d, *J* = 9.0 Hz, 1H, H-5′), 6.97 (d, *J* = 8.3 Hz, 1H, H-5), 7.13 (d, *J* = 1.7 Hz, 1H, H-2), 7.25 (dd, *J* = 1.7 Hz and 8.5 Hz, 1H, H-6), 7.43 (d, *J* = 15.4 Hz, 1H, H-α), 7.70 (d, *J* = 9.0 Hz, 1H, H-6′), 7.85 (d, *J* = 15.4 Hz, 1H, H-β), 13.32 (s, 1H, OH); ^13^C-NMR (CDCl_3_) δ 56.0, 56.1, 60.6, 102.6, 110.3, 114.8, 115.6, 117.6, 123.2, 125.7, 127.2, 136.6, 144.9, 146.6, 148.3, 158.1, 158.2, 192.2.

3,2′-Dihydroxy-4,3′,4′-trimethoxychalcone (**7k**): (0.24 g, 0.73 mmol, 73% yield); yellowish brown solid, 107–110 °C; ^1^H-NMR (CDCl_3_) δ 3.92 (s, 3H, OCH_3_), 3.95 (s, 3H, OCH_3_), 3.96 (s, 3H, OCH_3_), 5.73 (s, 1H, OH), 6.54 (d, *J* = 9.0 Hz, 1H, H-5′), 6.89 (d, *J* = 8.3 Hz, 1H, H-5), 7.14 (dd, *J* = 2.2 Hz and 8.3 Hz 1H, H-6), 7.30 (d, *J* = 2.2 Hz, 1H, H-2), 7.44 (d, *J* = 15.4 Hz, 1H, H-α), 7.68 (d, *J* = 9.0 Hz, 1H, H-6′), 7.82 (d, *J* = 15.4 Hz, 1H, H-β), 13.32 (s, 1H, OH); ^13^C-NMR (CDCl_3_) δ 55.9, 56.0, 60.5, 102.6, 110.3, 112.6, 115.4, 118.0, 122.9, 125.6, 128.1, 136.4, 144.3, 145.6, 148.7, 157.9, 158.0, 192.0.

2′-Hydroxy-3,4,3′,4′-tetramethoxychalcone (**7l**): (0.28 g, 0.80 mmol, 80% yield); orange-yellow solid, 117–120 °C; ^1^H-NMR (CDCl_3_) δ 3.93 (s, 3H, OCH_3_), 3.95 (s, 3H, OCH_3_), 3.96 (s, 3H, OCH_3_), 3.98 (s, 3H, OCH_3_), 6.55 (d, *J* = 9.3 Hz, 1H, H-5′), 6.92 (d, *J* = 8.3 Hz, 1H, H-5), 7.17 (d, *J* = 2.0 Hz, 1H, H-2), 7.27 (dd, *J* = 2.0 Hz and 8.3 Hz 1H, H-6), 7.45 (d, *J* = 15.4 Hz, 1H, H-α), 7.72 (d, *J* = 9.3 Hz, 1H, H-6′), 7.87 (d, *J* = 15.4 Hz, 1H, H-β), 13.34 (s, 1H, OH); ^13^C-NMR (CDCl_3_) δ 55.9, 55.9, 56.0, 60.5, 102.4, 109.9, 110.8, 115.3, 117.6, 123.1, 125.6, 127.3, 136.4, 144.5, 148.9, 151.2, 157.9, 158.0, 191.9.

### 3.7. The General Procedure for the Synthesis of Flavanones ***8a–l***

A solution of **7a**–**l** (1.0 mmol) and potassium fluoride (0.29 g, 5.0 mmol) in methanol (5 mL) was refluxed for 24 h. The water was added to a mixture solution and the mixture was extracted with EtOAc. The organic layer was washed with water and brine and dried over anhydrous MgSO_4_. The solvent was evaporated in vacuo and the residue was chromatographed on a preparative thin layer chromatography (hexane:toluene:EtOAc = 1:1:1) to produce flavanones **8a**–**l**.

7,4′-Dihydroxy-8-methoxyflavanone (**8a**): (0.28 g, 0.98 mmol, 98% yield); pale yellow solid, 195–200 °C; ^1^H-NMR (CD_3_OD) δ 2.72 (dd, *J* = 2.9 Hz and 17.1 Hz, 1H, H-3ax), 3.07 (dd, *J* = 12.9 Hz and 17.1 Hz, 1H, H-3eq), 3.79 (s, 3H, OCH_3_), 5.42 (dd, *J* = 2.9 Hz and 12.9 Hz, 1H, H-2), 6.55 (d, *J* = 8.8 Hz, 1H, H-6), 6.82 (td, *J* = 2.0 Hz and 8.5 Hz, 2H, H-3′ and H-5′), 7.35 (td, *J* = 1.7 Hz and 8.3 Hz, 2H, H-2′ and H-6′), 7.51 (d, *J* = 8.8 Hz, 1H, H-5); ^13^C-NMR (CD_3_OD) δ 44.8, 61.2, 81.3, 111.4, 115.9, 116.2, 123.7, 128.8, 131.0, 136.4, 157.4, 158.5, 158.7, 193.2; UV/Vis (1.5 × 10^−3^ M, DMSO); λ = 376.4 nm (ε, 1.2 × 10^2^).

7,3′,4′-Trihydroxy-8-methoxyflavanone (**8b**): (0.20 g, 0.65 mmol, 65% yield); yellowish brown solid, 188–193 °C; ^1^H-NMR (CD_3_OD) δ 2.72 (dd, *J* = 2.9 Hz and 17.1 Hz, 1H, H-3ax), 3.01 (dd, *J* = 12.9 Hz and 17.1 Hz, 1H, H-3eq), 3.81 (s, 3H, OCH_3_), 5.35 (dd, *J* = 2.9 Hz and 12.9 Hz, 1H, H-2), 6.54 (d, *J* = 8.8 Hz, 1H, H-6), 6.78 (d, *J* = 8.1 Hz, 1H, H-5′), 6.82 (dd, *J* = 1.7 Hz and 8.3 Hz, 1H, H-6′), 6.97 (d, *J* = 1.7 Hz, 1H, H-2′), 7.50 (d, *J* = 8.8 Hz, 1H, H-5); ^13^C-NMR (CD3OD) δ 44.9, 61.3, 81.3, 111.3, 114.5, 115.9, 116.1, 119.0, 123.6, 131.7, 136.4, 146.2, 146.6, 157.3, 158.4, 193.2; UV/Vis (2.6 × 10^−5^ M, CH_3_OH); λ = 392.0 nm (ε, 2.7 × 10^3^).

7,4′-Dihydroxy-8,3′-dimethoxyflavanone (**8d**): (0.28 g, 0.90 mmol, 90% yield); yellow solid, 193–195 °C; ^1^H-NMR (CD_3_OD) δ 2.67 (dd, *J* = 2.7 Hz and 16.8 Hz, 1H, H-3ax), 3.16 (dd, *J* = 12.7 Hz and 16.6 Hz, 1H, H-3eq), 3.69 (s, 3H, OCH_3_), 3.77 (s, 3H, OCH_3_), 5.46 (dd, *J* = 2.4 Hz and 12.9 Hz, 1H, H-2), 6.57 (d, *J* = 8.8 Hz, 1H, H-6), 6.79 (d, *J* = 8.8 Hz, 1H, H-6′), 6.93 (d, *J* = 8.8 Hz, 1H, H-5′), 7.11 (s, 1H, H-2′), 7.40 (d, *J* = 8.8 Hz, 1H, H-5); ^13^C-NMR (CD_3_OD) δ 43.2, 55.7, 60.2, 79.4, 110.4, 111.0, 114.4, 115.1, 119.3, 122.0, 129.9, 135.1, 146.7, 147.4, 155.7, 156.9, 190.2; UV/Vis (2.7 × 10^−5^ M, CH_3_OH); λ = 383.8 nm (ε, 1.7 × 10^4^).

7,3′-Dihydroxy-8,4′-dimethoxyflavanone (**8e**): (0.27 g, 0.85 mmol, 85% yield); yellowish brown solid, 180–185 °C; ^1^H-NMR (DMSO-*d*_6_) δ 2.69 (dd, *J* = 2.9 Hz and 16.8 Hz, 1H, H-3ax), 3.06 (dd, *J* = 12.5 Hz and 16.8 Hz, 1H, H-3eq), 3.71 (s, 3H, OCH_3_), 3.78 (s, 3H, OCH_3_), 5.48 (dd, *J* = 2.9 Hz Hz and 12.5 Hz, 1H, H-2), 6.58 (d, *J* = 8.8 Hz, 1H, H-6), 6.91 (dd, *J* = 1.7 Hz and 8.3 Hz, 1H, H-6′), 6.95 (d, *J* = 8.5 Hz, 1H, H-5′), 6.97 (d, *J* = 2.4 Hz, 1H, H-2′), 7.41 (d, *J* = 8.8 Hz, 1H, H-5); ^13^C-NMR (DMSO-*d*_6_) δ 43.1, 55.6, 60.1, 78.9, 110.2, 111.8, 113.8, 114.3, 117.3, 121.8, 131.4, 134.9, 146.2, 147.5, 155.4, 156.6, 189.8; UV/Vis (2.9 × 10^−5^ M, DMSO); λ = 363.6 nm (ε, 2.2 × 10^3^).

7-Hydroxy-8,3′,4′-trimethoxyflavanone (**8f**): (0.30 g, 0.90 mmol, 90% yield); yellow solid, 143–145 °C; ^1^H-NMR (CDCl_3_) δ 2.87 (dd, *J* = 2.9 Hz and 16.8 Hz, 1H, H-3ax), 3.06 (dd, *J* = 12.9 Hz and 16.8 Hz, 1H, H-3eq), 3.91 (s, 6H, OCH_3_), 3.95 (s, 3H, OCH_3_), 5.46 (dd, *J* = 2.7 Hz and 12.9 Hz, 1H, H-2), 6.59 (s, 1H, OH), 6.68 (d, *J* = 8.8 Hz, 1H, H-6), 6.91 (d, *J* = 8.8 Hz, 1H, H-5′), 7.02–7.03 (m, 2H, H-2′ and H-6′), 7.65 (d, *J* = 8.5 Hz, 1H, H-5); ^13^C-NMR (CDCl_3_) δ 44.2, 55.8, 61.1, 80.0, 109.1, 109.3, 110.9, 115.4, 118.4, 122.9, 130.9, 134.1, 148.8, 149.0, 154.2, 155.0, 190.2; UV/Vis (2.3 × 10^−5^ M, CHCl_3_); λ = 392.6 nm (ε, 1.4 × 10^3^).

4′-Hydroxy-7,8-dimethoxyflavanone (**8g**): (0.26 g, 0.87 mmol, 87% yield); yellow solid, 165–170 °C; ^1^H-NMR (CDCl_3_) δ 2.88 (dd, *J* = 2.9 Hz and 16.8 Hz, 1H, H-3ax), 3.07 (dd, *J* = 12.2 Hz and 16.8 Hz, 1H, H-eq), 3.88 (s, 3H, OCH_3_), 3.94 (s, 3H, OCH_3_), 5.45 (dd, *J* = 2.9 Hz and 12.2 Hz, 1H, H-2), 6.39 (s, 1H, OH), 6.67 (d, *J* = 9.0 Hz, 1H, H-6), 6.87 (d, *J* = 8.5 Hz, 2H, H-3′ and H-5′), 7.31 (d, *J* = 8.8 Hz, 2H, H-2′ and H-6′), 7.73 (d, *J* = 9.0 Hz, 1H, H-5); ^13^C-NMR (CDCl_3_) δ 44.0, 56.3, 61.1, 79.7, 105.7, 115.5, 116.0, 123.1, 127.8, 130.3, 136.7, 155.4, 156.2, 158.8, 191.6; UV/Vis (3.1 × 10^−5^ M, CHCl_3_); λ = 391.2 nm (ε, 1.2 × 10^3^).

3′,4′-Dihydroxy-7,8-dimethoxyflavanone (**8h**) (0.20 g, 0.63 mmol, 63% yield); yellowish brown solid, 175–177 °C; ^1^H-NMR (DMSO-*d*_6_) δ 2.71 (dd, *J* = 2.9 Hz and 16.8 Hz, 1H, H-3ax), 3.11 (dd, *J* = 12.5 Hz and 16.8 Hz, 1H, H-3eq), 3.69 (s, 3H, OCH_3_), 3.87 (s, 3H, OCH_3_), 5.45 (dd, *J* = 2.9 Hz and 12.5 Hz, 1H, H-2), 6.76–6.77 (m, 2H, H-6 and H-6′), 6.84 (d, *J* = 8.8 Hz, 1H, H-5′), 6.92 (d, *J* = 1.7 Hz, 1H, H-2′), 7.55 (d, *J* = 8.8 Hz, 1H, H-5); ^13^C-NMR (DMSO-*d*_6_) δ 43.2, 56.1, 60.2, 79.1, 105.8, 114.1, 115.1, 115.6, 117.6, 121.9, 129.6, 136.2, 144.9, 145.4, 154.7, 158.0, 190.3.

4′-Hydroxy-7,8,3′-trimethoxyflavanone (**8j**): (0.30 g, 0.91 mmol, 91% yield); yellow solid, 145–146 °C; ^1^H-NMR (CDCl_3_) δ 2.88 (dd, *J* = 2.9 Hz and 16.8 Hz, 1H, H-3ax), 3.06 (dd, *J* = 12.5 Hz and 16.8 Hz, 1H, H-3eq), 3.87 (s, 3H, OCH_3_), 3.91 (s, 3H, OCH_3_), 3.93 (s, 3H, OCH_3_), 5.44 (dd, *J* = 2.9 Hz and 12.5 Hz, 1H, H-2), 5.78 (s, 1H, OH), 6.67 (d, *J* = 8.8 Hz, 1H, H-6), 6.92–6.99 (m, 2H, H-5′ and H-6′), 7.02 (d, *J* = 1.5 Hz, 1H, H-2′), 7.71 (d, *J* = 9.0 Hz, 1H, H-5); ^13^C-NMR (CDCl_3_) δ 44.1, 55.8, 56.1, 60.9, 79.7, 105.4, 108.6, 114.2, 115.9, 119.1, 122.6, 130.4, 136.7, 145.6, 146.3, 155.0, 158.4, 190.6; UV/Vis (6.1 × 10^−4^ M, CHCl_3_); λ = 370.0 nm (ε, 1.9 × 10^3^).

3′-Hydroxy-7,8,4′-trimethoxyflavanone (**8k**): (0.30 g, 0.90 mmol, 90% yield); ^1^H-NMR (CDCl_3_) δ 2.85 (dd, *J* = 2.9 Hz and 16.8 Hz, 1H, H-3ax), 3.02 (dd, *J* = 12.5 Hz and 16.8 Hz, 1H, H-3eq), 3.87 (s, 3H, OCH_3_), 3.89 (s, 3H, OCH_3_), 3.93 (s, 3H, OCH_3_), 5.41 (dd, *J* = 2.9 Hz and 12.5 Hz, 1H, H-2), 5.94 (s, 1H, OH), 6.66 (d, *J* = 9.0 Hz, 1H, H-6), 6.87 (d, *J* = 8.3 Hz, 1H, H-5′), 6.95 (dd, *J* = 2.0 Hz and 8.3 Hz, 1H, H-6′), 7.08 (d, *J* = 2.0 Hz, 1H, H-2′), 7.70 (d, *J* = 9.0 Hz, 1H, H-5); ^13^C-NMR (CDCl_3_) δ 44.1, 55.9, 56.1, 60.9, 79.4, 105.4, 110.4, 112.4, 115.9, 117.7, 122.6, 131.6, 136.7, 145.5, 146.5, 155.0, 158.4, 190.7.

3′,4′,7,8-Tetramethoxyflavanone (**8l**): (0.16 g, 0.46 mmol, 46% yield); yellow solid, 141–143 °C; ^1^H-NMR (CDCl_3_) δ 2.90 (dd, *J* = 3.2 Hz and 16.8 Hz, 1H, H-3ax), 3.07 (dd, *J* = 12.2 Hz and 16.8 Hz, 1H, H-eq), 3.88 (s, 3H, OCH_3_), 3.90 (s, 3H, OCH_3_), 3.91 (s, 3H, OCH_3_), 3.94 (s, 3H, OCH_3_), 5.48 (dd, *J* = 2.9 Hz and 12.2 Hz, 1H, H-2), 6.67 (d, *J* = 9.0 Hz, 1H, H-6), 6.90 (d, *J* = 8.1 Hz, 1H, H-5′), 7.01–7.04 (m, 2H, H-2′ and H-6′), 7.71 (d, *J* = 8.8 Hz, 1H, H-5); ^13^C-NMR (CDCl_3_) δ 44.1, 55.8, 55.8, 56.1, 60.9, 79.5, 105.4, 109.2, 110.8, 116.0, 118.4, 122.6, 131.0, 136.7, 148.8, 148.9, 154.9, 158.4, 190.5; GC-MS 344 (M+, 44), 180 (35), 164 (100).

### 3.8. The General Procedure for the Synthesis of Chalcones ***9a–k*** by the Selective Deprotection of the 2′-Methoxymethyl Group

A solution of **6a**–**k** (1.0 mmol) and 1.5 M hydrochloric acid aqueous solution (5 mL) in THF (5 mL) was stirred at room temperature for 45 min. The mixture was extracted with Et_2_O. The organic layer was washed with water and brine and dried over anhydrous MgSO_4_. The solvent was evaporated in vacuo and the residue was chromatographed on a preparative thin layer chromatography (hexane:EtOAc = 3:2) to produce chalcones **9a**–**k**.

2′-Hydroxy-3′-methoxy-4,4′-di(methoxymethoxy)chalcone (**9a**): (0.29 g, 0.77 mmol, 77% yield); ^1^H-NMR (CDCl_3_) δ 3.50 (s, 3H, OCH_3_), 3.53 (s, 3H, OCH_3_), 3.94 (s, 3H, OCH_3_), 5.23 (s, 2H, OCH_2_), 5.32 (s, 2H, OCH_2_), 6.75 (d, *J* = 9.0 Hz, 1H, H-5′), 7.09 (d, *J* = 8.8 Hz, 2H, H-3 and H-5), 7.47 (d, *J* = 15.4 Hz, 1H, H-α), 7.61 (d, *J* = 8.8 Hz, 2H, H-2 and H-6), 7.64 (d, *J* = 9.0 Hz, 1H, H-6′), 7.88 (d, *J* = 15.4 Hz, 1H, H-β), 13.33 (s, 1H, OH); ^13^C-NMR (CDCl_3_) δ 56.1, 56.4, 60.6, 94.0, 94.5, 106.0, 116.0, 116.3, 117.9, 125.2, 128.1, 130.0, 144.3, 155.6, 158.2, 159.1, 192.1.

2′-Hydroxy-3′-methoxy-3,4,4′-tri(methoxymethoxy)chalcone (**9b**): (0.38 g, 0.87 mmol, 87% yield); orange solid, 84–88 °C; ^1^H-NMR (CDCl_3_) δ 3.53 (s, 3H, OCH_3_), 3.53 (s, 3H, OCH_3_), 3.56 (s, 3H, OCH_3_), 3.95 (s, 3H, OCH_3_), 5.30 (s, 2H, OCH_2_), 5.30 (s, 2H, OCH_2_), 5.32 (s, 2H, OCH_2_), 6.76 (d, *J* = 9.0 Hz, 1H, H-5′), 7.21 (d, *J* = 8.5 Hz, 1H, H-5), 7.29 (dd, *J* = 2.0 Hz and 8.5 Hz, 1H, H-6), 7.44 (d, *J* = 15.4 Hz, 1H, H-α), 7.48 (d, *J* = 2.0 Hz, 1H, H-2), 7.66 (d, *J* = 9.0 Hz, H-6′), 7.84 (d, *J* = 15.4 Hz, 1H, H-β), 13.31 (s, 1H, OH); ^13^C-NMR (CDCl_3_) δ 56.3, 56.3, 56.4, 60.7, 94.6, 95.0, 95.5, 106.2, 116.1, 116.1, 118.6, 124.1, 125.5, 129.0, 137.4, 144.3, 144.5, 147.3, 149.6, 155.8, 158.4, 192.3.

2′-Hydroxy-3′-methoxy-2,4,4′-tri(methoxymethoxy)chalcone (**9c**): (0.32 g, 0.73 mmol, 73% yield); ^1^H-NMR (CDCl_3_) δ 3.50 (s, 3H, OCH_3_), 3.53 (s, 3H, OCH_3_), 3.53 (s, 3H, OCH_3_), 3.94 (s, 3H, OCH_3_), 5.21 (s, 2H, OCH_2_), 5.29 (s, 2H, OCH_2_), 5.32 (s, 2H, OCH_2_), 6.75 (d, *J* = 9.0 Hz, 1H, H-5′), 6.76 (dd, *J* = 2.2 Hz and 8.5 Hz, 1H, H-5), 6.87 (d, *J* = 2.2 Hz, 1H, H-3), 7.59 (d, *J* = 15.6 Hz, 1H, H-α), 7.61 (d, *J* = 8.5 Hz, 1H, H-6), 7.64 (d, *J* = 9.3 Hz, 1H, H-6′), 8.21 (d, *J* = 15.4 Hz, 1H, H-β), 13.46 (s, 1H, OH); ^13^C-NMR (CDCl_3_) δ 56.2, 56.3, 56.4, 60.6, 94.1, 94.4, 94.5, 103.1, 105.9, 109.2, 116.1, 118.1, 118.3, 125.2, 129.8, 137.2, 139.8, 155.4, 157.6, 158.2, 160.3, 192.6.

2′-Hydroxy-3,3′-dimethoxy-4,4′-di(methoxymethoxy)chalcone (**9d**): (0.35 g, 0.87 mmol, 87% yield); ^1^H-NMR (CDCl_3_) δ 3.53 (s, 6H, OCH_3_), 3.95 (s, 3H, OCH_3_), 3.97 (s, 3H, OCH_3_), 5.30 (s, 2H, OCH_2_), 5.33 (s, 2H, OCH_2_), 6.77 (d, *J* = 9.3 Hz, 1H, H-5′), 7.17 (d, *J* = 1.7 Hz, 1H, H-2), 7.20 (d, *J* = 8.3 Hz, 1H, H-5), 7.25 (dd, *J* = 1.7 Hz and 8.3 Hz, 1H, H-6), 7.46 (d, *J* = 15.4 Hz, 1H, H-α), 7.67 (d, *J* = 9.3 Hz, 1H, H-6′), 7.86 (d, *J* = 15.4 Hz, 1H, H-β), 13.34 (s, 1H, OH); ^13^C-NMR (CDCl_3_) δ 55.9, 56.3, 56.4, 60.6, 94.4, 94.9, 105.9, 110.8, 115.5, 115.9, 118.1, 122.5, 125.2, 128.6, 137.2, 144.5, 148.7, 149.4, 155.6, 158.2, 192.0.

2′-Hydroxy-4,3′-dimethoxy-3,4′-di(methoxymethoxy)chalcone (**9e**): (0.26 g, 0.65 mmol, 65% yield); ^1^H-NMR (CDCl_3_) δ 3.53 (s, 3H, OCH_3_), 3.56 (s, 3H, OCH_3_), 3.95 (s, 6H, OCH_3_), 5.30 (s, 2H, OCH_2_), 5.33 (s, 2H, OCH_2_), 6.76 (d, *J* = 9.0 Hz, 1H, H-5′), 6.94 (d, *J* = 8.3 Hz, 1H, H-5), 7.31 (dd, *J* = 2.0 Hz and 8.3 Hz, 1H, H-6), 7.43 (d, *J* = 15.4 Hz, 1H, H-α), 7.49 (d, *J* = 2.2 Hz, 1H, H-2), 7.67 (d, *J* = 9.3 Hz, 1H, H-6′), 7.85 (d, *J* = 15.4 Hz, 1H, H-β), 13.36 (s, 1H, OH); ^13^C-NMR (CDCl_3_) δ 55.9, 56.2, 56.4, 60.6, 94.4, 95.3, 105.9, 111.3, 115.0, 115.9, 117.8, 124.5, 125.3, 127.4, 137.1, 144.5, 146.5, 151.9, 155.5, 158.2, 192.0.

2′-Hydroxy-3,4,3′-trimethoxy-4′-(methoxymethoxy)chalcone (**9f**): (0.36 g, 0.95 mmol, 95% yield); ^1^H-NMR (CDCl_3_) δ 3.53 (s, 3H, OCH_3_), 3.95 (s, 6H, OCH_3_), 3.97 (s, 3H, OCH_3_), 5.33 (s, 2H, OCH_2_), 6.76 (d, *J* = 9.0 Hz, 1H, H-5′), 6.92 (d, *J* = 8.5 Hz, H-5), 7.16 (d, *J* = 1.7 Hz, 1H, H-2), 7.26 (dd, *J* = 1.7 Hz and 8.3 Hz, 1H, H-6), 7.44 (d, *J* = 15.4 Hz, 1H, H-α), 7.67 (d, *J* = 9.0 Hz, 1H, H-6′), 7.87 (d, *J* = 15.4 Hz, 1H, H-β), 13.37 (s, 1H, OH); ^13^C-NMR (CDCl_3_) δ 55.8, 55.9, 56.4, 60.6, 94.4, 105.9, 109.9, 110.8, 115.9, 117.5, 123.1, 125.2, 127.3, 137.2, 144.7, 148.9, 151.3, 155.5, 158.2, 192.0.

2′-Hydroxy-3′,4′-dimethoxy-4-(methoxymethoxy)chalcone (**9g**): (0.32 g, 0.93 mmol, 93% yield); yellow solid, 92–95 °C; ^1^H-NMR (CDCl_3_) δ 3.50 (s, 3H, OCH_3_), 3.93 (s, 3H, OCH_3_), 3.96 (s, 3H, OCH_3_), 5.23 (s, 2H, OCH_2_), 6.54 (d, *J* = 9.0 Hz, 1H, H-5′), 7.09 (d, *J* = 8.8 Hz, 2H, H-3 and H-5), 7.48 (d, *J* = 15.4 Hz, 1H, H-α), 7.61 (d, *J* = 8.8 Hz, 2H, H-2 and H-6), 7.69 (d, *J* = 9.0 Hz, 1H, H-6′), 7.87 (d, *J* = 15.4 Hz, 1H, H-β), 13.31 (s, 1H, OH); ^13^C-NMR (CDCl_3_) δ 56.0, 56.1, 60.5, 94.0, 102.5, 115.4, 116.3, 117.9, 125.6, 128.1, 130.0, 136.4, 144.1, 157.9, 158.0, 159.0, 192.0.

2′-Hydroxy-3′,4′-dimethoxy-3,4-di(methoxymethoxy)chalcone (**9h**): (0.38 g, 0.93 mmol, 93% yield); ^1^H-NMR (CDCl_3_) δ 3.53 (s, 3H, OCH_3_), 3.56 (s, 3H, OCH_3_), 3.93 (s, 3H, OCH_3_), 3.96 (s, 3H, OCH_3_), 5.30 (s, 2H, OCH_2_), 5.31 (s, 2H, OCH_2_), 6.55 (d, *J* = 9.0 Hz, 1H, H-5′), 7.21 (d, *J* = 8.3 Hz, 1H, H-5), 7.29 (dd, *J* = 2.0 Hz and 8.5 Hz, 1H, H-6), 7.45 (d, *J* = 15.4 Hz, 1H, H-α), 7.48 (d, *J* = 2.0 Hz, 1H, H-2), 7.70 (d, *J* = 9.0 Hz, 1H, H-6′), 7.84 (d, *J* = 15.4 Hz, 1H, H-β), 13.28 (s, 1H, OH); ^13^C-NMR (CDCl_3_) δ 56.0, 56.2, 60.5, 94.9, 95.3, 102.5, 115.4, 115.8, 115.9, 118.4, 123.9, 125.7, 128.8, 136.4, 144.1, 147.1, 149.3, 157.9, 158.1, 191.9.

2′-Hydroxy-3′,4′-dimethoxy-2,4-di(methoxymethoxy)chalcone (**9i**): (0.38 g, 0.93 mmol, 93% yield); light yellow solid, 93–95 °C; ^1^H-NMR (CDCl_3_) δ 3.50 (s, 3H, OCH_3_), 3.53 (s, 3H, OCH_3_), 3.93 (s, 3H, OCH_3_), 3.96 (s, 3H, OCH_3_), 5.22 (s, 2H, OCH_2_), 5.29 (s, 2H, OCH_2_), 6.54 (d, *J* = 9.0 Hz, 1H, H-5′), 6.76 (dd, *J* = 2.4 Hz and 8.8 Hz, 1H, H-5), 6.87 (d, *J* = 2.2 Hz, 1H, H-3), 7.59 (d, *J* = 15.6 Hz, 1H, H-α), 7.62 (d, *J* = 8.8 Hz, 1H, H-6), 7.69 (d, *J* = 9.0 Hz, 1H, H-6′), 8.22 (d, *J* = 15.4 Hz, 1H, H-β), 13.43 (s, 1H, OH); ^13^C-NMR (CDCl_3_) δ 56.0, 56.2, 56.3, 60.5, 94.0, 94.4, 102.4, 103.0, 109.1, 115.5, 118.1, 118.3, 125.5, 129.6, 136.4, 139.5, 157.6, 157.9, 157.9, 160.2, 192.4.

2′-Hydroxy-3,3′,4′-trimethoxy-4-(methoxymethoxy)chalcone (**9j**): (0.34 g, 0.90 mmol, 90% yield); ^1^H-NMR (CDCl_3_) δ 3.53 (s, 3H, OCH_3_), 3.93 (s, 3H, OCH_3_), 3.96 (s, 3H, OCH_3_), 3.97 (s, 3H, OCH_3_), 5.30 (s, 2H, OCH_2_), 6.54 (d, *J* = 9.0 Hz, 1H, H-5′), 7.17 (d, *J* = 2.0 Hz, 1H, H-2), 7.20 (d, *J* = 8.5 Hz, 1H, H-5), 7.24 (dd, *J* = 1.7 Hz and 8.3 Hz, 1H, H-6), 7.46 (d, *J* = 15.4 Hz, 1H, H-α), 7.70 (d, *J* = 9.0 Hz, 1H, H-6′), 7.85 (d, *J* = 15.4 Hz, 1H, H-β), 13.30 (s, 1H, OH); ^13^C-NMR (CDCl_3_) δ 55.9, 56.0, 56.3, 60.5, 94.9, 102.5, 111.0, 115.4, 115.6, 118.2, 122.4, 125.6, 128.7, 136.4, 144.3, 148.7, 149.5, 157.9, 158.1, 191.9.

2′-Hydroxy-4,3′,4′-trimethoxy-3-(methoxymethoxy)chalcone (**9k**): (0.36 g, 0.95 mmol, 95% yield); ^1^H-NMR (CDCl_3_) δ 3.57 (s, 3H, OCH_3_), 3.93 (s, 3H, OCH_3_), 3.95 (s, 3H, OCH_3_), 3.96 (s, 3H, OCH_3_), 5.30 (s, 2H, OCH_2_), 6.55 (d, *J* = 9.3 Hz, 1H, H-5′), 6.94 (d, *J* = 8.3 Hz, 1H, H-5), 7.31 (dd, *J* = 2.2 Hz and 8.3 Hz, 1H, H-6), 7.44 (d, *J* = 15.4 Hz, 1H, H-α), 7.49(d, *J* = 2.2 Hz, 1H, H-2), 7.71 (d, *J* = 9.0 Hz, 1H, H-6′), 7.85 (d, *J* = 15.4 Hz, 1H, H-β), 13.32 (s, 1H, OH); ^13^C-NMR (CDCl_3_) δ 55.9, 56.0, 56.2, 60.5, 95.4, 102.5, 111.4, 115.0, 115.4, 117.8, 124.5, 125.7, 127.5, 136.4, 144.3, 146.5, 151.9, 157.9, 158.0, 191.9.

### 3.9. The General Procedure for the Synthesis of Flavonols ***10a–l***

To a solution of **7l** and **9a**–**k** (1.0 mmol) in methanol (25 mL), a 4 M sodium hydroxide aqueous solution (0.3 mL, 1.2 mmol) and a 30% hydrogen peroxide solution (0.5 mL, 5.0 mmol) were added at room temperature. After being stirred at room temperature for 12 h, the mixture was poured into ice and a 2 M hydrochloric acid aqueous solution. The precipitate was filtered, washed with water, and dried in vacuo to produce flavonols **10a**–**l**.

8-Methoxy-7,4′-di(methoxymethoxy)flavonol (**10a**): (0.20 g, 0.51 mmol, 51% yield); ^1^H-NMR (CDCl_3_) δ 3.51 (s, 3H, OCH_3_), 3.56 (s, 3H, OCH_3_), 4.07 (s, 3H, OCH_3_), 5.26 (s, 2H, OCH_2_), 5.36 (s, 2H, OCH_2_), 7.20 (d, *J* = 9.0 Hz, 2H, H-3′ and H-5′), 7.26 (d, *J* = 9.0 Hz, 1H, H-6), 7.93 (d, *J* = 9.0 Hz, 1H, H-5), 8.26 (d, *J* = 9.0 Hz, 2H, H-2′ and H-6′); ^13^C-NMR (CDCl_3_) δ 56.1, 56.5, 61.6, 94.1, 95.1, 113.6, 116.0, 116.1, 116.5, 120.5, 124.7, 129.2, 129.2, 137.2, 137.7, 144.8, 149.7, 153.9, 158.4, 172.7.

8-Methoxy-7,3′,4′-tri(methoxymethoxy)flavonol (**10b**): (0.16 g, 0.36 mmol, 36% yield); ^1^H-NMR (CDCl_3_) δ 3.55 (s, 3H, OCH_3_), 3.56 (s, 3H, OCH_3_), 3.58 (s, 3H, OCH_3_), 4.08 (s, 3H, OCH_3_), 5.33 (s, 2H, OCH_2_), 5.34 (s, 2H, OCH_2_), 5.36 (s, 2H, OCH_2_), 7.09 (s, 1H, OH), 7.26 (d, *J* = 9.0 Hz, 1H, H-6), 7.32 (d, *J* = 8.8 Hz, 1H, H-5′), 7.92 (d, *J* = 9.3 Hz, 1H, H-5), 7.99 (dd, *J* = 2.0 Hz and 8.8 Hz, 1H, H-6′), 8.18 (d, *J* = 2.0 Hz, 1H, H-2′); ^13^C-NMR (CDCl_3_) δ 56.2, 56.2, 56.4, 61.5, 94.9, 95.0, 95.5, 109.7, 113.5, 115.8, 116.2, 120.3, 122.5, 125.1, 137.1, 137.5, 143.9, 146.7, 148.5, 149.6, 153.7, 172.4.

8-Methoxy-7,2′,4′-tri(methoxymethoxy)flavonol (**10c**): (0.15 g, 0.33 mmol, 33% yield); ^1^H-NMR (CDCl_3_) δ 3.48 (s, 3H, OCH_3_), 3.51 (s, 3H, OCH_3_), 3.55 (s, 3H, OCH_3_), 3.98 (s, 3H, OCH_3_), 5.20 (s, 2H, OCH_2_), 5.23 (s, 2H, OCH_2_), 5.34 (s, 2H, OCH_2_), 6.43 (s, 1H, OH), 6.85 (dd, *J* = 2.2 Hz and 8.5 Hz, 1H, H-6′), 7.00 (d, *J* = 2.2 Hz, 1H, H-2′), 7.27 (d, *J* = 9.0 Hz, 1H, H-6), 7.54 (d, *J* = 8.5 Hz, 1H, H-5′), 7.96 (d, *J* = 9.0 Hz, 1H, H-5); ^13^C-NMR (CDCl_3_) δ 56.1, 56.4, 61.4, 94.1, 94.9, 95.0, 104.0, 108.8, 113.4, 114.0, 117.0, 120.3, 131.4, 137.5, 137.8, 145.1, 150.2, 153.4, 156.2, 159.8, 172.4.

8,3′-Dimethoxy-7,4′-di(methoxymethoxy)flavonol (**10d**): (0.11 g, 0.26 mmol, 26% yield); ^1^H-NMR (CDCl_3_) δ 3.55 (s, 3H, OCH_3_), 3.56 (s, 3H, OCH_3_), 4.00 (s, 3H, OCH_3_), 4.07 (s, 3H, OCH_3_), 5.33 (s, 2H, OCH_2_), 5.36 (s, 2H, OCH_2_), 7.00 (s, 1H, OH), 7.27 (d, *J* = 9.0 Hz, 1H, H-6), 7.32 (d, *J* = 8.5 Hz, 1H, H-5′), 7.90 (dd, *J* = 2.0 Hz and 8.8 Hz, 1H, H-6′), 7.93 (d, *J* = 9.0 Hz, 1H, H-5), 7.95 (d, *J* = 2.0 Hz, 1H, H-2′); ^13^C-NMR (CDCl_3_) δ 55.8, 56.2, 56.5, 61.5, 95.0, 110.8, 113.5, 115.5, 116.2, 120.4, 120.8, 125.1, 137.1, 137.4, 144.1, 147.7, 149.1, 149.5, 153.7, 172.4.

8,4′-Dimethoxy-7,3′-di(methoxymethoxy)flavonol (**10e**): (0.27 g, 0.65 mmol, 65% yield); ^1^H-NMR (CDCl_3_) δ 3.56 (s, 3H, OCH_3_), 3.58 (s, 3H, OCH_3_), 3.98 (s, 3H, OCH_3_), 4.08 (s, 3H, OCH_3_), 5.34 (s, 2H, OCH_2_), 5.36 (s, 2H, OCH_2_), 6.98 (s, 1H, OH), 7.06 (d, *J* = 8.8 Hz, 1H, H-5′), 7.26 (d, *J* = 9.0 Hz, 1H, H-6), 7.92 (d, *J* = 9.0 Hz, 1H, H-5), 8.04 (dd, *J* = 2.2 Hz and 8.5 Hz, 1H, H-6′), 8.17 (d, *J* = 2.2 Hz, 1H, H-2′); ^13^C-NMR (CDCl_3_) δ 55.9, 56.2, 56.4, 61.5, 95.0, 95.5, 111.3, 113.4, 115.3, 116.3, 120.3, 122.7, 123.8, 137.0, 137.5, 144.0, 146.1, 149.5, 151.0, 153.7, 172.4.

8,3′,4′-Trimethoxy-7-(methoxymethoxy)flavonol (**10f**): (0.15 g, 0.38 mmol, 38% yield); light yellow solid, 163–166 °C; ^1^H-NMR (CDCl_3_) δ 3.56 (s, 3H, OCH_3_), 3.98 (s, 3H, OCH_3_), 4.01 (s, 3H, OCH_3_), 4.08 (s, 3H, OCH_3_), 5.37 (s, 2H, OCH_2_), 7.00 (s, 1H, OH), 7.05 (d, *J* = 8.5 Hz, 1H, H-6), 7.28 (d, *J* = 7.6 Hz, 1H, H-5′), 7.93 (s, 1H, H-2′), 7.94 (d, *J* = 8.8 Hz, 1H, H-5), 7.96 (dd, *J* = 2.0 Hz and 8.1Hz, 1H, H-6′); ^13^C-NMR (CDCl_3_) δ 55.7, 55.8, 56.5, 61.5, 94.9, 110.1, 110.7, 113.3, 116.2, 120.4, 121.0, 123.6, 136.9, 137.3, 144.2, 148.4, 149.5, 150.2, 153.6, 172.3.

7,8-Dimethoxy-4′-(methoxymethoxy)flavonol (**10g**): (0.16 g, 0.45 mmol, 45% yield); ^1^H-NMR (CDCl_3_) δ 3.51 (s, 3H, OCH_3_), 4.02 (s, 3H, OCH_3_), 4.04 (s, 3H, OCH_3_), 5.27 (s, 2H, OCH_2_), 6.94 (s, 1H, OH), 7.07 (d, *J* = 9.0 Hz, 1H, H-6), 7.20 (d, *J* = 9.0 Hz, 2H, H-3′ and H-5′), 7.97 (d, *J* = 9.0 Hz, 1H, H-5), 8.26 (d, *J* = 9.0 Hz, 2H, H-2′ and H-6′); ^13^C-NMR (CDCl_3_) δ 56.0, 56.4, 61.4, 94.0, 109.8, 115.4, 115.9, 120.5, 124.6, 129.0, 136.7, 144.3, 149.4, 156.0, 158.1, 172.5.

7,8-Dimethoxy-3′,4′-di(methoxymethoxy)flavonol (**10h**): (0.12 g, 0.28 mmol, 28% yield); ^1^H-NMR (CDCl_3_) δ 3.55 (s, 3H, OCH_3_), 3.58 (s, 3H, OCH_3_), 4.02 (s, 3H, OCH_3_), 4.05 (s, 3H, OCH_3_), 5.33 (s, 2H, OCH_2_), 5.34 (s, 2H, OCH_2_), 6.97 (s, 1H, OH), 7.07 (d, *J* = 9.3 Hz, 1H, H-6), 7.33 (d, *J* = 8.8 Hz, 1H, H-5′), 7.96 (d, *J* = 9.0 Hz, 1H, H-5), 7.98 (dd, *J* = 2.2 Hz and 8.8 Hz, 1H, H-6′), 8.18 (d, *J* = 2.2 Hz, 1H, H-2′); ^13^C-NMR (CDCl_3_) δ 56.2, 56.2, 56.4, 61.4, 94.9, 95.5, 109.8, 115.3, 115.7, 115.8, 120.5, 122.5, 125.2, 136.5, 136.9, 143.8, 146.7, 148.4, 149.4, 156.1, 172.5.

7,8-Dimethoxy-2′,4′-di(methoxymethoxy)flavonol (**10i**): (0.07 g, 0.17 mmol, 17% yield); ^1^H-NMR (CDCl_3_) δ 3.48 (s, 3H, OCH_3_), 3.51 (s, 3H, OCH_3_), 3.96 (s, 3H, OCH_3_), 4.01 (s, 3H, OCH_3_), 5.20 (s, 2H, OCH_2_), 5.23 (s, 2H, OCH_2_), 6.42 (s, 1H, OH), 6.85 (dd, *J* = 2.2 Hz and 8.8 Hz, 1H, H-6′), 7.00 (d, *J* = 2.2 Hz, 1H, H-2′), 7.08 (d, *J* = 9.0 Hz, 1H, H-6), 7.54 (d, *J* = 8.8 Hz, 1H, H-5′), 8.00 (d, *J* = 9.0 Hz, 1H, H-5); ^13^C-NMR (CDCl_3_) δ 56.1, 56.4, 61.3, 94.1, 94.8, 104.0, 108.8, 109.6, 114.0, 116.1, 120.5, 131.4, 136.5, 137.6, 145.0, 150.0, 155.7, 156.2, 159.8, 172.5.

7,8,3′-Trimethoxy-4′-(methoxymethoxy)flavonol (**10j**): (0.22 g, 0.56 mmol, 56% yield); ^1^H-NMR (CDCl_3_) δ 3.55 (s, 3H, OCH_3_), 4.00 (s, 3H, OCH_3_), 4.02 (s, 3H, OCH_3_), 4.05 (s, 3H, OCH_3_), 5.33 (s, 2H, OCH_2_), 7.00 (s, 1H, OH), 7.08 (d, *J* = 9.0 Hz, 1H, H-6), 7.32 (d, *J* = 8.8 Hz, 1H, H-5′), 7.90 (dd, *J* = 2.0 Hz and 8.8 Hz, 1H, H-6′), 7.95 (d, *J* = 2.0 Hz, 1H, H-2′), 7.97 (d, *J* = 9.0 Hz, 1H, H-5); ^13^C-NMR (CDCl_3_) δ 55.8, 56.2, 56.4, 61.3, 94.9, 109.8, 110.8, 115.3, 115.4, 120.6, 120.8, 125.1, 136.4, 136.9, 144.0, 147.7, 149.0, 149.4, 156.1, 172.5.

7,8,4′-Trimethoxy-3′-(methoxymethoxy)flavonol (**10k**): (0.24 g, 0.61 mmol, 61% yield); ^1^H-NMR (CDCl_3_) δ 3.58 (s, 3H, OCH_3_), 3.98 (s, 3H, OCH_3_), 4.02 (s, 3H, OCH_3_), 4.06 (s, 3H, OCH_3_), 5.34 (s, 2H, OCH_2_), 7.00 (s, 1H, OH), 7.06 (d, *J* = 8.5 Hz, 1H, H-5′), 7.07 (d, *J* = 9.0 Hz, 1H, H-6), 7.96 (d, *J* = 9.0 Hz, 1H, H-5), 8.04 (dd, *J* = 2.2 Hz and 8.8 Hz, 1H, H-6′), 8.18 (d, *J* = 2.0 Hz, 1H, H-2′); ^13^C-NMR (CDCl_3_) δ 56.0, 56.4, 56.6, 61.6, 95.7, 109.9, 111.4, 115.5, 116.0, 120.7, 122.9, 124.0, 136.6, 137.0, 144.2, 146.2, 149.5, 151.1, 156.2, 172.6.

7,8,3′,4′-Tetramethoxyflavonol (**10l**): (0.12 g, 0.33 mmol, 33% yield); pale yellow solid, 215–217 °C; ^1^H-NMR (CDCl_3_) δ 3.98 (s, 3H, OCH_3_), 4.00 (s, 3H, OCH_3_), 4.02 (s, 3H, OCH_3_), 4.05 (s, 3H, OCH_3_), 7.01 (s, 1H, OH), 7.04 (d, *J* = 8.5 Hz, 1H, H-5′), 7.08 (d, *J* = 9.0 Hz, 1H, H-6), 7.92 (d, *J* = 2.0 Hz, 1H, H-2′), 7.95 (dd, *J* = 2.0 Hz and 8.1 Hz, 1H, H-6′), 7.97 (d, *J* = 8.8 Hz, 1H, H-5); ^13^C-NMR (CDCl_3_) δ 55.7, 55.8, 56.4, 61.3, 109.7, 110.2, 110.7, 115.3, 120.5, 121.0, 123.7, 136.4, 136.7, 144.2, 148.5, 149.3, 150.2, 156.0, 172.4; UV/Vis (2.1 × 10^−5^ M, CHCl_3_); λ = 363.6 nm (ε, 2.8 × 10^4^).

### 3.10. The General Procedure for the Deprotection of Flavonols ***10a–k***

A solution of **10a**–**k** (1.0 mmol) in methanol (5 mL) and 3 M hydrochloric acid (5 mL) was refluxed for 1 h. The mixture was extracted with EtOAc. The organic layer was washed with water and brine and dried over anhydrous MgSO_4_. The solvent was evaporated in vacuo and the residue was chromatographed on a preparative thin layer chromatography (hexane:EtOAc = 2:3) to produce flavonols **11a**–**k**.

7,4′-Dihydroxy-8-methoxyflavonol (**11a**): (0.18 g, 0.45 mmol, 80% yield); pale yellowish brown solid, 263–267 °C; ^1^H-NMR (DMSO-*d*_6_) δ 3.93 (s, 3H, OCH_3_), 6.96 (d, *J* = 9.0 Hz, 2H, H-3′ and H-5′), 6.98 (d, *J* = 8.8 Hz, 1H, H-6), 7.68 (d, *J* = 8.8 Hz, 1H, H-5), 8.06 (d, *J* = 8.8 Hz, 2H, H-2′ and H-6′), 9.14 (s, 1H, OH), 10.07 (s, 1H, OH); ^13^C-NMR (DMSO-*d*_6_) δ 60.8, 114.6, 114.9, 115.3, 119.8, 122.1, 128.8, 134.4, 136.8, 144.7, 149.1, 154.1, 158.5, 171.8; UV/Vis (2.8 × 10^−5^ M, CH_3_OH); λ = 358.5 nm (ε, 1.8 × 10^4^).

7,3′,4′-Trihydroxy-8-methoxyflavonol (**11b**): (0.16 g, 0.49 mmol, 98% yield); yellow solid, 257–258 °C; ^1^H-NMR (DMSO-*d*_6_) δ 3.94 (s, 3H, OCH_3_), 6.91 (d, *J* = 8.5 Hz, 1H, H-5′), 6.98 (d, *J* = 9.0 Hz, 1H, H-6), 7.59 (dd, *J* = 2.2 and 8.5 Hz, 1H, H-6′), 7.67 (d, *J* = 8.8 Hz, 1H, H-5), 7.72 (d, *J* = 2.2 Hz, 1H, H-2′), 9.10 (s, 1H, OH), 9.35 (s, 1H, OH), 9.52 (s, 1H, OH), 10.51 (s, 1H, OH); ^13^C-NMR (DMSO-*d*_6_) δ 60.9, 114.5, 114.6, 114.9, 115.4, 119.4, 119.8, 122.4, 134.4, 136.8, 144.7, 144.8, 147.0, 149.1, 154.0, 171.7; UV/Vis (2.5 × 10^−5^ M, CH_3_OH); λ = 366.0 nm (ε, 2.1 × 10^4^).

7,2′,4′-Trihydroxy-8-methoxyflavonol (**11c**): (0.06 g, 0.18 mmol, 36% yield); pale green-yellow solid, 265 °C (decomp.); ^1^H-NMR (DMSO-*d*_6_) δ 3.90 (s, 3H, OCH_3_), 6.17 (d, *J* = 1.7 Hz, 1H, H-3′), 6.31 (dd, *J* = 2.0 Hz and 8.8 Hz, 1H, H-5′), 6.95 (d, *J* = 8.8 Hz, 1H, H-6), 7.52 (d, *J* = 8.3 Hz, 1H, H-6′), 7.68 (d, *J* = 8.8 Hz, 1H, H-5), 9.46 (s, 1H, OH), 10.32 (s, 1H, OH); ^13^C-NMR (DMSO-*d*_6_) δ 60.8, 104.8, 106.1, 113.2, 114.1, 115.0, 119.7, 127.9, 134.4, 142.9, 146.8, 149.4, 153.1, 159.8, 160.6, 175.9; UV/Vis (2.7 × 10^−5^ M, CH_3_OH); λ = 395.0 nm (ε, 1.5 × 10^4^).

7,4′-Dihydroxy-8,3′-dimethoxyflavonol (**11d**): (0.14 g, 0.43 mmol, 85% yield); pale yellow solid, 281–282 °C; ^1^H-NMR (DMSO-*d*_6_) δ 3.85 (s, 3H, OCH_3_), 3.95 (s, 3H, OCH_3_), 6.97 (d, *J* = 8.5 Hz, 1H, H-5′), 6.99 (d, *J* = 8.8 Hz, 1H, H-6), 7.68 (d, *J* = 8.8 Hz, 1H, H-5), 7.74 (dd, *J* = 2.0 Hz and 8.5 Hz, 1H, H-6′), 7.78 (d, *J* = 2.0 Hz, 1H, H-2′), 9.25 (s, 1H, OH), 9.75 (s, 1H, OH), 10.56 (s, 1H, OH); ^13^C-NMR (DMSO-*d*_6_) δ 55.6, 60.9, 111.0, 114.7, 114.9, 115.5, 119.9, 121.2, 122.5, 134.4, 137.0, 144.6, 147.2, 148.2, 149.2, 154.2, 171.9; UV/Vis (2.7 × 10^−5^ M, acetone); λ = 353.0 nm (ε, 1.8 × 10^4^).

7,3′-Dihydroxy-8,4′-dimethoxyflavonol (**11e**): (0.14 g, 0.43 mmol, 86% yield); yellow solid, 241–244 °C; ^1^H-NMR (DMSO-*d*_6_) δ 3.86 (s, 3H, OCH_3_), 3.95 (s, 3H, OCH_3_), 7.00 (d, *J* = 8.8 Hz, 1H, H-6), 7.13 (d, *J* = 8.5 Hz, 1H, H-5′), 7.69 (d, *J* = 8.8 Hz, 1H, H-5), 7.71 (dd, *J* = 2.2 Hz and 8.5 Hz, 1H, H-6′), 7.74 (d, *J* = 2.2 Hz, 1H, H-2′), 9.21 (s, 1H, OH), 9.39 (s, 1H, OH), 10.54 (s, 1H, OH); ^13^C-NMR (DMSO-*d*_6_) δ 55.5, 60.9, 111.7, 114.2, 114.7, 114.9, 119.1, 119.8, 123.9, 134.4, 137.2, 144.2, 146.0, 148.7, 149.1, 154.1, 171.8; UV/Vis (2.8 × 10^−5^ M, CH_3_OH); λ = 361.5 nm (ε, 2.2 × 10^4^).

7-Hydroxy-8,3′,4′-trimethoxyflavonol (**11f**): (0.07 g, 0.19 mmol, 38% yield); ocher solid, 202–206 °C; ^1^H-NMR (DMSO-*d*_6_) δ 3.85 (s, 3H, OCH_3_), 3.86 (s, 3H, OCH_3_), 3.96 (s, 3H, OCH_3_), 7.00 (d, *J* = 8.8 Hz, 1H, H-6), 7.18 (d, *J* = 8.5 Hz, 1H, H-5′), 7.69 (d, *J* = 8.8 Hz, 1H, H-5), 7.80 (d, *J* = 2.0 Hz, 1H, H-2′), 7.85 (dd, *J* = 2.0 Hz and 8.5 Hz, 1H, H-6′), 9.29 (s, 1H, OH), 10.56 (s, 1H, OH); ^13^C-NMR (DMSO-*d*_6_) δ 55.4, 55.6, 60.8, 110.3, 111.5, 114.8, 114.9, 119.9, 120.8, 123.8, 134.4, 137.4, 144.1, 148.2, 149.2, 149.8, 154.3, 171.9; UV/Vis (2.4 × 10^−5^ M, CHCl_3_); λ = 359.0 nm (ε, 1.9 × 10^4^).

4′-Hydroxy-7,8,dimethoxyflavonol (**11g**): (0.09 g, 0.29 mmol, 57% yield); pale yellow solid, 235–236 °C; ^1^H-NMR (DMSO-*d*_6_) δ 3.93 (s, 3H, OCH_3_), 3.96 (s, 3H, OCH_3_), 6.97 (d, *J* = 8.8 Hz, 2H, H-3′ and H-5′), 7.27 (d, *J* = 9.0 Hz, 1H, H-6), 7.83 (d, *J* = 9.0 Hz, 1H, H-5), 8.08 (d, *J* = 8.8 Hz, 2H, H-2′ and H-6′), 9.26 (s, 1H, OH), 10.12 (s, 1H, OH); ^13^C-NMR (DMSO-*d*_6_) δ 56.5, 61.0, 110.4, 115.3, 116.1, 119.9, 122.0, 129.0, 135.9, 136.9 145.3, 148.4, 155.5, 158.7, 171.9; UV/Vis (2.7 × 10^−5^ M, CH_3_OH); λ = 361.5 nm (ε, 2.5 × 10^4^).

3′,4′-Dihydroxy-7,8-dimethoxyflavonol (**11h**): (0.13 g, 0.40 mmol, 79% yield); yellow ocher solid, 261–263 °C; ^1^H-NMR (DMSO-*d*_6_) δ 3.84 (s, 3H, OCH_3_), 3.94 (s, 3H, OCH_3_), 6.37 (dd, *J* = 2.0 Hz and 8.3 Hz, 1H, H-5′), 6.42 (d, *J* = 2.0 Hz, 1H, H-3′), 7.25 (d, *J* = 9.0 Hz, 1H, H-6), 7.26 (d, *J* = 8.3 Hz, 1H, H-6′), 7.83 (d, *J* = 9.0 Hz, 1H, H-5), 9.68 (s, 1H, OH), 9.78 (s, 1H, OH); ^13^C-NMR (DMSO-*d*_6_) δ 56.4, 60.9, 102.9, 106.6, 109.5, 110.3, 116.8, 119.9, 131.6, 136.0, 137.4, 147.5, 149.2, 155.3, 156.7, 160.1, 172.1; UV/Vis (2.5 × 10^−5^ M, CH_3_OH); λ = 369.0 nm (ε, 2.4 × 10^4^).

2′,4′-Dihydroxy-7,8,3′-trimethoxyflavonol (**11i**): (0.07 g, 0.22 mmol, 43% yield); red-clay solid, 192–196 °C; ^1^H-NMR (DMSO-*d*_6_) δ 3.95 (s, 3H, OCH_3_), 3.96 (s, 3H, OCH_3_), 6.92 (d, *J* = 8.5 Hz, 1H, H-5′), 7.26 (d, *J* = 9.0 Hz, 1H, H-6), 7.62 (dd, *J* = 2.0 Hz and 8.5 Hz, 1H, H-6′), 7.75 (d, *J* = 2.0 Hz, 1H, H-2′), 7.82 (d, *J* = 9.0 Hz, 1H, H-5), 9.22 (s, 1H, OH), 9.40 (s, 1H, OH), 9.56 (s, 1H, OH); ^13^C-NMR (DMSO-*d*_6_) δ 56.4, 61.1, 110.3, 114.6, 115.4, 116.0, 119.6, 119.9, 122.3, 135.8, 136.9, 144.8, 145.2, 147.2, 148.3, 155.4, 171.8.

4′-Hydroxy-7,8,3′-trimethoxyflavonol (**11j**): (0.08 g, 0.24 mmol, 48% yield); ocher solid, 177–181 °C; ^1^H-NMR (CDCl_3_) δ 4.01 (s, 3H, OCH_3_), 4.02 (s, 3H, OCH_3_), 4.05 (s, 3H, OCH_3_), 6.01 (s, 1H, OH), 7.00 (s, 1H, OH), 7.07 (d, *J* = 9.0 Hz, 1H, H-6), 7.09 (d, *J* = 8.3 Hz, 1H, H-5′), 7.88 (dd, *J* = 2.0 Hz and 8.5 Hz, 1H, H-6′), 7.93 (d, *J* = 2.0 Hz, 1H, H-2′), 7.97 (d, *J* = 9.0 Hz, 1H, H-5); ^13^C-NMR (CDCl_3_) δ 55.8, 56.4, 61.3, 109.7, 109.9, 114.4, 115.3, 120.5, 121.4, 123.2, 136.4, 136.6, 144.3, 146.1, 147.1, 149.3, 156.0, 172.4; UV/Vis (2.8 × 10^−5^ M, CHCl_3_); λ = 362.5 nm (ε, 2.3 × 10^4^).

3′-Hydroxy-7,8,4′-trimethoxyflavonol (**11k**): (0.09 g, 0.27 mmol, 53% yield); pale yellow solid, 231–234 °C; ^1^H-NMR (DMSO-*d*_6_) δ 3.86 (s, 3H, OCH_3_), 3.95 (s, 3H, OCH_3_), 3.97 (s, 3H, OCH_3_), 7.13 (d, *J* = 8.5 Hz, 1H, H-5′), 7.27 (d, *J* = 9.0 Hz, 1H, H-6), 7.72 (dd, *J* = 2.2 Hz and 8.5 Hz, 1H, H-6′), 7.75 (d, *J* = 2.2 Hz, 1H, H-2′), 7.83 (d, *J* = 9.0 Hz, 1H, H-5), 9.33 (s, 1H, OH), 9.42 (s, 1H, OH); ^13^C-NMR (DMSO-*d*_6_) δ 55.5, 56.4, 61.0, 110.3, 111.7, 114.2, 116.0, 119.3, 119.9, 123.8, 135.8, 137.3, 144.7, 146.0, 148.4, 148.8, 155.5, 171.9; UV/Vis (2.3 × 10^−5^ M, CHCl_3_); λ = 360.5 nm (ε, 2.6 × 10^4^).

### 3.11. The General Procedure for the Preparation of 4-Chloroacetylpyrogallols ***12a,b***

Chloroacetyl chloride (4.3 mL, 54.0 mmol) was added to a suspension of anhydrous aluminum chloride (8.00 g, 60.0 mmol) and 1,2-dichloroethane (100 mL) was added under an argon atmosphere at room temperature. A solution of **1a**,**b** (30.0 mmol) in 1,2-dichloroethane (30 mL) was added to the mixture and the reaction mixture was stirred at room temperature for 12 h. The mixture was poured into ice and a 2 M HCl solution and extracted with CHCl3. The organic layer was washed with water and dried over anhydrous MgSO_4_. The solvent was evaporated in vacuo and the residue was chromatographed on silica gel with CHCl3-Et_2_O (9:1) to produce **12a**,**b**.

1-Chloroacetyl-2,4-dihydroxy-3-methoxybenzene (**12a**): (3.77 g, 17.4 mmol, 58% yield); ^1^H-NMR (CDCl_3_) δ 4.00 (s, 3H, OCH_3_), 4.64 (s, 2H, CH2), 6.56 (d, *J* = 9.0 Hz, 1H, H-5), 6.57 (s, 1H, OH), 7.41 (d, *J* = 9.0 Hz, 1H, H-6), 12.24 (s, 1H, OH); ^13^C-NMR (CDCl_3_) δ 44.8, 60.9, 107.2, 112.2, 126.3, 134.3, 155.9, 156.8, 195.2.

1-Chloroacetyl-2-hydroxy-3,4-dimethoxybenzene (**12b**): (3.74 g, 16.2 mmol, 54% yield); colorless solid, 155–158 °C; ^1^H-NMR (CDCl_3_) δ 3.89 (s, 3H, OCH_3_), 3.95 (s, 3H, OCH_3_), 4.65 (s, 2H, CH2), 6.54 (d, *J* = 9.0 Hz, 1H, H-5), 7.49 (d, *J* = 9.0 Hz, 1H, H-6), 11.85 (s, 1H, OH); ^13^C-NMR (CDCl_3_) δ 45.0, 56.2, 60.7, 103.5, 112.7, 126.1, 136.6, 157.2, 159.1, 195.0.

### 3.12. The General Procedure for the Preparation of Benzofuranones ***13a,b***

A solution of **12a**,**b** (20.0 mmol) and sodium acetate (6.56 g, 80.0 mmol or 3.28 g, 40.0 mmol) in methanol (100 mL) was refluxed for 2 h. Water was added to the mixture and extracted with Et_2_O. The organic layer was washed with brine and dried over anhydrous MgSO_4_. The solvent was evaporated in vacuo and the residue was chromatographed on silica gel with CHCl_3_-Et_2_O (9:1) to produce **13a**,**b**.

6-Hydroxy-7-methoxy-3(2*H*)-benzofuranone (**13a**): (2.77 g, 15.4 mmol, 77% yield); ^1^H-NMR (DMSO-*d*_6_) δ 3.89 (s, 3H, OCH_3_), 4.76 (s, 2H, CH_2_), 6.85 (d, *J* = 8.5 Hz, 1H, H-5), 7.12 (d, *J* = 8.5 Hz, 1H, H-4), 9.37 (s, 1H, OH); ^13^C-NMR (DMSO-*d*_6_) δ 56.5, 75.2, 107.4, 113.7, 115.5, 131.7, 154.6, 162.1, 198.0.

6,7-Dimethoxy-3(*2H*)-benzofuranone (**13b**): (3.22 g, 16.6 mmol, 83% yield); reddish yellow solid, 119–123 °C; ^1^H-NMR (CDCl_3_) δ 3.97 (s, 3H, OCH_3_), 4.01 (s, 3H, OCH_3_), 4.67 (s, 2H, CH_2_), 6.72 (d, *J* = 8.5 Hz, 1H, H-5), 7.41 (d, *J* = 8.5 Hz, 1H, H-4); ^13^C-NMR (CDCl_3_) δ 56.7, 61.0, 75.5, 107.3, 116.3, 119.1, 134.2, 159.4, 166.0, 197.8.

### 3.13. The General Procedure for the Protection of ***13a*** with a Chloromethyl Methyl Ether

A solution of **13a** (0.90 g, 5.0 mmol) in DMF (5 mL) was added to a suspension of sodium hydride (60% in mineral oil, 0.24 g, 6.0 mmol) in DMF (15 mL) at 0 °C. After being stirred at room temperature for 30 min, a chloromethyl methyl ether (0.57 mL, 7.5 mmol) was added to the mixture at 0 °C. After being stirred at room temperature for 6 h, Et_2_O (20 mL) was added to the mixture. The reaction mixture was poured into ice water (200 mL). The mixture was extracted with Et_2_O. The organic layer was washed with water and brine and dried over anhydrous MgSO_4_. The solvent was evaporated in vacuo and the residue was chromatographed on silica gel with CHCl_3_-Et_2_O (9:1) to produce **13c**.

7-Methoxy-6-(methoxymethoxy)-3(*2H*)-benzofuranone (**13c**): (0.81 g, 3.6 mmol, 72% yield); dark brown solid, 203–210 °C; ^1^H-NMR (CDCl_3_) δ 3.53 (s, 3H, OCH_3_), 4.03 (s, 3H, OCH_3_), 4.68 (s, 2H, OCH_2_), 5.32 (s, 2H, OCH_2_), 6.94 (d, *J* = 8.5 Hz, 1H, H-5), 7.37 (d, *J* = 8.5 Hz, 1H, H-4); ^13^C-NMR (CDCl_3_) δ 56.6, 61.0, 75.4, 95.0, 110.8, 117.0, 118.8, 156.9, 166.3, 197.9.

### 3.14. The General Procedure for the Synthesis of Aurones ***14a–l***

Aluminum oxide (basic, 2.00 g, 19.6 mmol) was added to a solution of benzofuranones **13b**,**c** (1.0 mmol) and benzaldehydes **5a**–**f** (1.2 mmol) in dichloromethane (5 mL). The mixture was thoroughly stirred for 2 days at room temperature. The suspension was filtered off and the residue was washed with CHCl_3_. The filtrate was concentrated in vacuo and the residue was chromatographed on a preparative thin layer chromatography (CHCl_3_:Et_2_O = 9:1) to produce (*Z*)-aurones **14a**–**l**.

(*Z*)-7-Methoxy-6,4′-di(methoxymethoxy)aurone (**14a**): (0.32 g, 0.86 mmol, 86% yield); reddish yellow solid, 92–94 °C; ^1^H-NMR (CDCl_3_) δ 3.50 (s, 3H, OCH_3_), 3.55 (s, 3H, OCH_3_), 4.19 (s, 3H, OCH_3_), 5.24 (s, 2H, OCH_2_), 5.33 (s, 2H, OCH_2_), 6.84 (s, 1H, H-10), 7.03 (d, *J* = 8.5 Hz, 1H, H-5), 7.12 (d, *J* = 9.0 Hz, 2H, H-3′ and H-5′), 7.48 (d, *J* = 8.3 Hz, 1H, H-4), 7.87 (d, *J* = 8.8 Hz, 2H, H-2′ and H-6′); ^13^C-NMR δ (CDCl_3_) 56.1, 56.5, 61.0, 93.9, 95.1, 111.7, 112.2, 116.3, 117.6, 119.0, 125.7, 132.7, 134.6, 146.1, 155.9, 157.4, 158.1, 182.7.

(*Z*)-7-Methoxy-6,3′,4′-tri(methoxymethoxy)aurone (**14b**): (0.23 g, 0.54 mmol, 54% yield); light yellow solid, 85–86 °C; ^1^H-NMR (CDCl_3_) δ 3.54 (s, 3H, OCH_3_), 3.55 (s, 3H, OCH_3_), 3.56 (s, 3H, OCH_3_), 4.21 (s, 3H, OCH_3_), 5.32 (s, 4H, OCH_2_), 5.34 (s, 2H, OCH_2_), 6.82 (s, 1H, H-10), 7.04 (d, *J* = 8.5 Hz, 1H, H-5), 7.24 (d, *J* = 8.5 Hz, 1H, H-5′), 7.47 (dd, *J* = 2.0 Hz and 8.3 Hz, 1H, H-6′), 7.48 (d, *J* = 8.5 Hz, 1H, H-4), 7.88 (d, *J* = 2.0 Hz, 1H, H-2′); ^13^C-NMR (CDCl_3_) δ 56.1, 56.2, 56.5, 61.0, 94.7, 95.0, 95.1, 111.5, 112.2, 115.7, 117.5, 118.4, 118.9, 126.2, 126.5, 134.5, 146.1, 146.8, 148.3, 155.8, 157.3, 182.8.

(*Z*)-7-Methoxy-6,2′,4′-tri(methoxymethoxy)aurone (**14c**): (0.29 g, 0.67 mmol, 67% yield); yellow solid, 105–110 °C; ^1^H-NMR (CDCl_3_) δ 3.50 (s, 3H, OCH_3_), 3.52 (s, 3H, OCH_3_), 3.67 (s, 3H, OCH_3_), 3.99 (s, 3H, OCH_3_), 5.22 (s, 2H, OCH_2_), 5.27 (s, 2H, OCH_2_), 5.33 (s, 2H, OCH_2_), 6.78 (dd, *J* = 2.2 Hz and 9.0 Hz, 1H, H-5′) 6.81, (d, *J* = 8.8 Hz, 1H, H-5), 6.87 (d, *J* = 2.2 Hz, 1H, H-3′), 7.38 (s, 1H, H-10), 7.58 (d, *J* = 8.5 Hz, 1H, H-4), 8.27 (d, *J* = 8.8 Hz, 1H, H-6′); ^13^C-NMR (CDCl_3_) δ 56.2, 56.3, 56.5, 61.0, 93.9, 94.4, 95.1, 102.7, 106.5, 109.1, 111.4, 115.5, 117.8, 118.9, 132.4, 133.6, 146.2, 155.6, 157.2, 157.6, 159.5, 182.8.

(*Z*)-7,3′-Dimethoxy-6,4′-di(methoxymethoxy)aurone (**14d**): (0.28 g, 0.69 mmol, 69% yield); reddish yellow solid, 95–100 °C; ^1^H-NMR (CDCl_3_) δ 3.54 (s, 3H, OCH_3_), 3.55 (s, 3H, OCH_3_), 3.98 (s, 3H, OCH_3_), 4.18 (s, 3H, OCH_3_), 5.32 (s, 2H, OCH_2_), 5.34 (s, 2H, OCH_2_), 6.84 (s, 1H, H-10), 7.04 (d, *J* = 8.5 Hz, 1H, H-5), 7.24 (d, *J* = 8.5 Hz, 1H, H-5′), 7.44 (dd, *J* = 2.0 Hz and 8.5 Hz, 1H, H-6′), 7.50 (d, *J* = 8.5 Hz, 1H, H-4), 7.60 (d, *J* = 2.0 Hz, 1H, H-2′); ^13^C-NMR (CDCl_3_) δ 55.6, 56.2, 56.5, 60.8, 94.8, 95.1, 111.6, 112.4, 113.5, 115.4, 117.5, 119.2, 125.2, 126.2, 134.4, 146.2, 147.7, 149.2, 155.9, 157.5, 182.7.

(*Z*)-7,4′-Dimethoxy-6,3′-di(methoxymethoxy)aurone (**14e**): (0.34 g, 0.85 mmol, 85% yield); light yellow solid, 130–133 °C; ^1^H-NMR (CDCl_3_) δ 3.55 (s, 3H, OCH_3_), 3.56 (s, 3H, OCH_3_), 3.95 (s, 3H, OCH_3_), 4.20 (s, 3H, OCH_3_), 5.30 (s, 2H, OCH_2_), 5.33 (s, 2H, OCH_2_), 6.82 (s, 1H, H-10), 6.97 (d, *J* = 8.5 Hz, 1H, H-5′), 7.03 (d, *J* = 8.5 Hz, 1H, H-5), 7.48 (dd, *J* = 2.0 Hz and 8.5 Hz, 1H, H-6′), 7.48 (d, *J* = 8.5 Hz, 1H, H-4), 7.89 (d, *J* = 2.0 Hz, 1H, H-2′); ^13^C-NMR (CDCl_3_) δ 55.8, 56.1, 56.5, 61.0, 95.2, 95.3, 111.4, 111.7, 112.5, 117.7, 118.1, 118.9, 125.0, 126.9, 134.6, 146.0, 146.4, 151.0, 155.8, 157.3, 182.7.

(*Z*)-7,3′,4′-Trimethoxy-6-(methoxymethoxy)aurone (**14f**): (0.12 g, 0.31 mmol, 31% yield); light yellow solid, 157–161 °C; ^1^H-NMR (CDCl_3_) δ 3.55 (s, 3H, OCH_3_), 3.95 (s, 3H, OCH_3_), 3.98 (s, 3H, OCH_3_), 4.18 (s, 3H, OCH_3_), 5.34 (s, 2H, OCH_2_), 6.84 (s, 1H, H-10), 6.95 (d, *J* = 8.3 Hz, 1H, H-5′), 7.04 (d, *J* = 8.5 Hz, 1H, H-5), 7.44 (dd, *J* = 1.7 Hz and 8.5 Hz, 1H, H-6′), 7.49 (d, *J* = 8.3 Hz, 1H, H-4), 7.60 (d, *J* = 1.7 Hz, 1H, H-2′); ^13^C-NMR (CDCl_3_) δ 55.7, 55.9, 56.6, 60.9, 95.2, 111.0, 111.7, 112.9, 113.1, 117.7, 119.3, 125.1, 125.7, 134.6, 146.1, 148.8, 150.5, 156.1, 157.6, 182.9.

(*Z*)-6,7-Dimethoxy-4′-(methoxymethoxy)aurone (**14g**): (0.31 g, 0.89 mmol, 89% yield); ^1^H-NMR (CDCl_3_) δ 3.50 (s, 3H, OCH_3_), 3.99 (s, 3H, OCH_3_), 4.18 (s, 3H, OCH_3_), 5.24 (s, 2H, OCH_2_), 6.80 (d, *J* = 8.5 Hz, 1H, H-5), 6.83 (s, 1H, H-10), 7.12 (d, *J* = 8.5 Hz, 2H, H-3′ and H-5′), 7.52 (d, *J* = 8.5 Hz, 1H, H-4), 7.87 (d, *J* = 8.8 Hz, 2H, H-2′ and H-6′); ^13^C-NMR (CDCl_3_) δ 56.2, 56.8, 61.1, 94.2, 108.0, 112.1, 116.6, 117.1, 119.5, 126.0, 132.9, 133.9, 146.6, 157.4, 158.4, 158.6, 183.0.

(*Z*)-6,7-Dimethoxy-3′,4′-di(methoxymethoxy)aurone (**14h**): (0.31 g, 0.78 mmol, 78% yield); yellow solid, 133–135 °C; ^1^H-NMR (CDCl_3_) δ 3.53 (s, 3H, OCH_3_), 3.56 (s, 3H, OCH_3_), 3.99 (s, 3H, OCH_3_), 4.19 (s, 3H, OCH_3_), 5.31 (s, 4H, OCH_2_), 6.80 (s, 1H, H-10), 6.80 (d, *J* = 8.5 Hz, 1H, H-5), 7.24 (d, *J* = 8.3 Hz, 1H, H-5′), 7.47 (dd, *J* = 2.0 Hz and 8.3 Hz, 1H, H-6′), 7.52 (d, *J* = 8.5 Hz, 1H, H-4), 7.87 (d, *J* = 2.0 Hz, 1H, H-2′); ^13^C-NMR (CDCl_3_) δ 56.1, 56.2, 56.7, 61.0, 94.8, 95.2, 107.7, 111.9, 115.8, 116.7, 118.6, 119.2, 126.4, 126.5, 133.6, 146.3, 146.9, 148.4, 157.0, 158.3, 182.7.

(*Z*)-6,7-Dimethoxy-2′,4′-di(methoxymethoxy)aurone (**14i**): (0.36 g, 0.89 mmol, 89% yield); light yellow solid, 115–120 °C; ^1^H-NMR (CDCl_3_) δ 3.50 (s, 3H, OCH_3_), 3.52 (s, 3H, OCH_3_), 3.99 (s, 3H, OCH_3_), 4.16 (s, 3H, OCH_3_), 5.22 (s, 2H, OCH_2_), 5.27 (s, 2H, OCH_2_), 6.80 (d, *J* = 8.5 Hz, 1H, H-5), 6.83 (dd, *J* = 2.2 Hz and 8.8 Hz, 1H, H-5′), 6.88 (d, *J* = 2.2 Hz, 1H, H-3′), 7.38 (s, 1H, H-10), 7.52 (d, *J* = 8.3 Hz, 1H, H-4), 8.26 (d, *J* = 8.5 Hz, 1H, H-6′); ^13^C-NMR (CDCl_3_) δ 56.1, 56.3, 56.6, 60.9, 94.0, 94.5, 102.8, 106.2, 107.6, 109.2, 115.7, 117.0, 119.2, 132.4, 133.6, 146.4, 156.9, 157.6, 158.1, 159.5, 182.7.

(*Z*)-6,7,3′-Trimethoxy-4′-(methoxymethoxy)aurone (**14j**): (0.30 g, 0.81 mmol, 81% yield); yellow solid, 158–162 °C; ^1^H-NMR (CDCl_3_) δ 3.53 (s, 3H, OCH_3_), 3.98 (s, 3H, OCH_3_), 4.00 (s, 3H, OCH_3_), 4.16 (s, 3H, OCH_3_), 5.31 (s, 2H, OCH_2_), 6.81 (d, *J* = 8.1 Hz, 1H, H-5), 6.82 (s, 1H, H-10), 7.23 (d, *J* = 8.5 Hz, 1H, H-5′), 7.43 (dd, *J* = 2.0 Hz and 8.5 Hz, 1H, H-6′), 7.53 (d, *J* = 8.5 Hz, 1H, H-4), 7.59 (d, *J* = 1.7 Hz, 1H, H-2′); ^13^C-NMR (CDCl_3_) δ 55.7, 56.2, 56.7, 60.8, 94.9, 107.8 112.2, 113.6, 115.5, 116.7, 119.4, 125.2, 126.3, 133.5, 146.4, 147.7, 149.2, 157.2, 158.4, 182.6.

(*Z*)-6,7,4′-Trimethoxy-3′-(methoxymethoxy)aurone (**14k**): (0.29 g, 0.78 mmol, 78% yield); yellow solid, 162–167 °C; ^1^H-NMR (CDCl_3_) δ 3.55 (s, 3H, OCH_3_), 3.94 (s, 3H, OCH_3_), 4.00 (s, 3H, OCH_3_), 4.19 (s, 3H, OCH_3_), 5.30 (s, 2H, OCH_2_), 6.80 (d, *J* = 8.3 Hz, 1H, H-5), 6.80 (s, 1H, H-10), 6.97 (d, *J* = 8.3 Hz, 1H, H-5′), 7.48 (dd, *J* = 2.0 Hz and 8.5 Hz, 1H, H-6′), 7.51 (d, *J* = 8.5 Hz, 1H, H-4), 7.88 (d, *J* = 2.2 Hz, 1H, H-2′); ^13^C-NMR (CDCl_3_) δ 56.0, 56.3, 56.8, 61.1, 95.4, 107.9 111.6, 112.3, 117.0, 118.3, 119.3, 125.2, 126.9, 133.8, 146.3, 146.5, 151.1, 157.1, 158.4, 182.8.

(*Z*)-6,7,3′4′-Tetramethoxyaurone (**14l**): (0.27 g, 0.80 mmol, 80% yield); yellow solid, 156–157 °C; ^1^H-NMR (CDCl_3_) δ 3.95 (s, 3H, OCH_3_), 3.98 (s, 3H, OCH_3_), 3.99 (s, 3H, OCH_3_), 4.16 (s, 3H, OCH_3_), 6.80 (d, *J* = 8.5 Hz, 1H, H-5), 6.82 (s, 1H, H-10), 6.95 (d, *J* = 8.3 Hz, 1H, H-5′), 7.44 (dd, *J* = 2.0 Hz and 8.5 Hz, 1H, H-6′), 7.53 (d, *J* = 8.3 Hz, 1H, H-4), 7.60 (d, *J* = 2.0 Hz, 1H, H-2′); ^13^C-NMR (CDCl_3_) δ 55.6, 55.8, 56.6, 60.8, 107.8, 110.9, 112.4, 113.0, 116.8, 119.4, 125.0, 125.5, 133.5, 146.2, 148.7, 150.3, 157.1, 158.4, 182.6; UV/Vis (2.2 × 10^−5^ M, CHCl_3_); λ = 406.8 nm (ε, 2.3 × 10^4^).

### 3.15. The General Procedure for the Deprotection of ***14a–k***

A solution of **14a**–**k** (1.0 mmol) in methanol (5 mL) and 3 M hydrochloric acid (5 mL) was refluxed for 1 h. The mixture was extracted with EtOAc. The organic layer was washed with water and brine and dried over anhydrous MgSO_4_. The solvent was evaporated in vacuo and the residue was chromatographed on a preparative thin layer chromatography (hexane:EtOAc = 2:3) to produce aurones **15a**–**k**.

(*Z*)-6,4′-Dihydroxy-7-methoxyaurone (**15a**): (0.18 g, 0.63 mmol, 63% yield); yellow brown solid, 240–241 °C; ^1^H-NMR (CDCl_3_:CD_3_OD = 1:1) δ 4.15 (s, 3H, OCH_3_), 6.75 (d, *J* = 8.5 Hz, 1H, H-5), 6.80 (s, 1H, H-10), 6.90 (d, *J* = 8.5 Hz, 2H, H-3′ and H-5′), 7.37 (d, *J* = 8.3 Hz, 1H, H-4), 7.81 (d, *J* = 8.8 Hz, 2H, H-2′ and H-6); ^13^C-NMR (CDCl_3_:CD_3_OD = 1:1) δ 61.1, 113.8, 114.2, 116.0, 116.5, 120.1, 124.2, 132.8, 133.9, 146.7, 158.5, 158.8, 160.0, 183.8; UV/Vis (3.0 × 10^−5^ M, CH_3_OH); λ = 394.6 nm (ε, 2.6 × 10^4^).

(*Z*)-6,3′,4′-Trihydroxy-7-methoxyaurone (**15b**): (0.27 g, 0.91 mmol, 91% yield); reddish ocher solid, 235–238 °C; ^1^H-NMR (DMSO-*d*_6_) δ 4.04 (s, 3H, OCH_3_), 6.70 (s, 1H, H-10), 6.79 (d, *J* = 8.3 Hz, 1H, H-5), 6.87 (d, *J* = 8.3 Hz, 1H, H-5′), 7.27 (dd, *J* = 2.0 Hz and 8.3 Hz, 1H, H-6′), 7.35 (d, *J* = 8.3 Hz, 1H, H-4), 7.46 (d, *J* = 1.7 Hz, 1H, H-2′), 9.45 (s, 1H, OH), 9.84 (s, 1H, OH), 10.96 (s, 1H, OH); ^13^C-NMR (DMSO-*d*_6_) δ 60.8, 112.3, 113.3, 114.7, 115.9, 117.8, 119.3, 123.2, 124.6, 132.1, 145.3, 145.4, 148.0, 157.6, 157.8, 181.1; UV/Vis (2.9 × 10^−5^ M, DMSO); λ = 411.2 nm (ε, 2.1 × 10^4^).

(*Z*)-6,2′,4′-Trihydroxy-7-methoxyaurone (**15c**): (0.25 g, 0.84 mmol, 84% yield); ocher solid, 288–290 °C; ^1^H-NMR (DMSO-*d*_6_) δ 3.94 (s, 3H, OCH_3_), 6.43 (d, *J* = 2.0 Hz, 1H, H-3′), 6.45 (dd, *J* = 2.2 Hz and 9.3 Hz, 1H, H-5′), 6.96 (d, *J* = 8.5 Hz, 1H, H-5), 7.12 (s, 1H, H-10), 7.26 (d, *J* = 8.5 Hz, 1H, H-4), 8.16 (d, *J* = 8.3 Hz, 1H, H-6′), 9.82 (s, 1H, OH), 10.16 (s, 1H, OH), 10.42 (s, 1H, OH); ^13^C-NMR (DMSO-*d*_6_) δ 56.6, 102.1, 106.3, 108.3, 108.3, 110.6, 114.6, 116.1, 131.3, 132.8, 145.0, 153.3, 154.5, 159.0, 160.8, 181.9; UV/Vis (2.8 × 10^−5^ M, DMSO); λ = 428.2 nm (ε, 3.3 × 10^4^).

(*Z*)-6,4′-Dihydroxy-7,3′-dimethoxyaurone (**15d**): (0.24 g, 0.75 mmol, 75% yield); yellow solid, 218–220 °C; ^1^H-NMR (DMSO-*d*_6_) δ 3.87 (s, 3H, OCH_3_), 4.04 (s, 3H, OCH_3_), 6.79 (d, *J* = 8.5 Hz, 1H, H-5), 6.79 (s, 1H, H-10), 6.92 (d, *J* = 8.3 Hz, 1H, H-5′), 7.36 (d, *J* = 8.5 Hz, 1H, H-4), 7.42 (dd, *J* = 1.7 Hz and 8.3 Hz, 1H, H-6′), 7.63 (d, *J* = 1.7 Hz, 1H, H-2′); ^13^C-NMR (DMSO-*d*_6_) δ 55.6, 60.7, 112.3, 113.5, 114.3, 114.7, 116.0, 119.5, 123.4, 125.9, 132.1, 145.6, 147.7, 148.9, 157.8, 157.9, 181.2; UV/Vis (2.4 × 10^−5^ M, DMSO); λ = 407.2 nm (ε, 2.9 × 10^4^).

(*Z*)-6,3′-Dihydroxy-7,4′-dimethoxyaurone (**15e**): (0.31 g, 0.97 mmol, 97% yield); yellow brown solid, 241–243 °C; ^1^H-NMR (DMSO-*d*_6_) δ 3.82 (s, 3H, OCH_3_), 4.01 (s, 3H, OCH_3_), 6.70 (s, 1H, H-10), 6.77 (d, *J* = 8.5 Hz, 1H, H-5), 7.05 (d, *J* = 8.5 Hz, 1H, H-5′), 7.33 (d, *J* = 8.3 Hz, 1H, H-4), 7.37 (dd, *J* = 1.5 Hz and 8.5 Hz, 1H, H-6′), 7.45 (d, *J* = 1.5 Hz, 1H, H-2′), 9.44 (s, 1H, OH), 10.97 (s, 1H, OH); ^13^C-NMR (DMSO-*d*_6_) δ 55.7, 60.8, 111.7, 112.1, 113.4, 114.5, 117.3, 119.4, 124.2, 124.5, 132.1, 145.7, 146.4, 149.5, 157.8, 157.9, 181.2; UV/Vis (2.7 × 10^−5^ M, DMSO); λ = 405.4 nm (ε, 2.1 × 10^4^).

(*Z*)-6-Hydroxy-7,3′,4′-trimethoxyaurone (**15f**): (0.21 g, 0.65 mmol, 65% yield); yellow solid, 204–205 °C; ^1^H-NMR (CDCl_3_) δ 3.95 (s, 3H, OCH_3_), 3.96 (s, 3H, OCH_3_), 4.27 (s, 3H, OCH_3_), 6.65 (s, 1H, OH), 6.82 (d, *J* = 8.5 Hz, 1H, H-5), 6.83 (s, 1H, H-10), 6.94 (d, *J* = 8.5 Hz, 1H, H-5′), 7.42 (dd, *J* = 2.0 Hz and 8.3 Hz, 1H, H-6′), 7.46 (d, *J* = 8.3 Hz, 1H, H-4), 7.51 (d, *J* = 2.0 Hz, H-2′); ^13^C-NMR (CDCl_3_) δ 55.7, 55.8, 60.6, 111.0, 111.7, 112.6, 113.0, 116.3, 119.7, 124.9, 125.4, 130.9, 146.0, 148.7, 150.4, 154.9, 115.9, 182.1; UV/Vis (2.4 × 10^−5^ M, CHCl_3_); λ = 400.0 nm (ε, 2.6 × 10^4^).

(*Z*)-4′-Hydroxy-6,7-dimethoxyaurone (**15g**): (0.27 g, 0.89 mmol, 89% yield); dark yellow solid, 230–231 °C; ^1^H-NMR (DMSO-*d*_6_) δ 4.00 (s, 3H, OCH_3_), 4.17 (s, 3H, OCH_3_), 5.76 (s, 1H, OH), 6.81 (d, *J* = 8.3 Hz, 1H, H-5), 6.84 (s, 1H, H-10), 6.95 (d, *J* = 8.3 Hz, 2H, H-3′ and H-5′), 7.54 (d, *J* = 8.5 Hz, 1H, H-4), 7.83 (d, *J* = 8.3 Hz, 2H, H-2′ and H-6′); ^13^C-NMR (DMSO-*d*_6_) δ 56.6, 60.6, 108.7, 112.0, 114.9, 116.0, 119.0, 119.1, 122.7, 133.1, 154.1, 156.5, 158.3, 159.2, 181.3; UV/Vis (3.0 × 10^−5^ M, DMSO); λ = 404.2 nm (ε, 2.5 × 10^4^).

(*Z*)-3′,4′-Dihydroxy-6,7-dimethoxyaurone (**15h**): (0.16 g, 0.51 mmol, 51% yield); dark yellow solid, 219–220 °C; ^1^H-NMR (CD_3_OD) δ 3.97 (s, 3H, OCH_3_), 4.08 (s, 3H, OCH_3_), 6.71 (s, 1H, H-10), 6.84 (d, *J* = 8.3 Hz, 1H, H-5), 6.94 (d, *J* = 8.5 Hz, 1H, H-5′), 7.26 (dd, *J* = 2.2 Hz and 8.3 Hz, 1H, H-6′), 7.46 (d, *J* = 8.5 Hz, 1H, H-4), 7.46 (d, *J* = 2.2 Hz, 1H, H-2′); ^13^C-NMR (CD_3_OD) δ 57.2, 61.5, 109.7, 115.0, 116.4, 117.6, 118.8, 120.3, 125.1, 126.3, 135.0, 146.4, 147.0, 149.3, 158.7, 160.5, 184.3; UV/Vis (2.5 × 10^−5^ M, CH_3_OH); λ = 408.2 nm (ε, 2.6 × 10^4^).

(*Z*)-2′,4′-Dihydroxy-6,7-dimethoxyaurone (**15i**): (0.25 g, 0.78 mmol, 78% yield); reddish clay solid, 245 °C (decomp.); ^1^H-NMR (DMSO-*d*_6_) δ 3.94 (s, 3H, OCH_3_), 4.02 (s, 3H, OCH_3_), 6.43 (s, 1H, H-10), 6.44 (d, J = 7.1 Hz, 1H, H-5), 7.01 (d, *J* = 8.3 Hz, 1H, H-5′), 7.14 (s, 1H, H-3′), 7.48 (d, *J* = 8.3 Hz, 1H, H-4), 7.98 (d, *J* = 8.5 Hz, 1H, H-6′), 10.17 (s, 1H, OH), 10.36 (s, 1H, OH); ^13^C-NMR (DMSO-*d*_6_) δ 56.8, 60.7, 102.3, 106.8, 108.5, 108.9, 110.4, 116.4, 119.0, 132.2, 133.3, 144.7, 156.5, 158.2, 159.2, 161.0, 181.2; UV/Vis (2.4 × 10^−5^ M, DMSO); λ = 424.6 nm (ε, 2.9 × 10^4^).

(*Z*)-4′-Hydroxy-6,7,3′-trimethoxyaurone (**15j**): (0.27 g, 0.81 mmol, 81% yield); dark yellow solid, 171–176 °C; ^1^H-NMR (CDCl_3_) δ 3.97 (s, 3H, OCH_3_), 3.99 (s, 3H, OCH_3_), 4.16 (s, 3H, OCH_3_), 6.13 (s, 1H, OH), 6.80 (d, *J* = 8.3 Hz, 1H, H-5), 6.81 (s, 1H, H-10), 6.99 (d, *J* = 8.3 Hz, 1H, H-5′), 7.39 (dd, *J* = 1.7 Hz and 8.3 Hz, 1H, H-6′), 7.52 (d, *J* = 8.5 Hz, 1H, H-4), 7.56 (d, *J* = 1.7 Hz, 1H, H-2′); ^13^C-NMR (CDCl_3_) δ 55.7, 56.6, 60.7, 107.8, 112.7, 112.7, 114.7, 116.8, 119.3, 124.5, 126.1, 133.5, 146.0, 146.3, 147.3, 157.0, 158.3, 182.5; UV/Vis (2.8 × 10^−5^ M, CHCl_3_); λ = 405.4 nm (ε, 1.7 × 10^4^).

(*Z*)-3′-Hydroxy-6,7,4′-trimethoxyaurone (**15k**): (0.30 g, 0.91 mmol, 91% yield); dark yellow solid, 196–200 °C; ^1^H-NMR (CDCl_3_) δ 3.94 (s, 3H, OCH_3_), 3.98 (s, 3H, OCH_3_), 4.20 (s, 3H, OCH_3_), 5.86 (s, 1H, OH), 6.78 (s, 1H, H-10), 6.79 (d, *J* = 9.3 Hz, 1H, H-5), 6.92 (d, *J* = 8.3 Hz, 1H, H-5′), 7.39 (dd, *J* = 2.0 Hz and 8.3 Hz, 1H, H-6′), 7.51 (d, *J* = 8.5 Hz, 1H, H-4), 7.57 (d, *J* = 2.2 Hz, 1H, H-2′); ^13^C-NMR (CDCl_3_) δδ 56.0, 56.8, 61.1, 107.9, 110.6, 112.4, 116.7, 116.9, 119.3, 124.7, 125.6, 133.8, 145.6, 146.4, 148.0, 157.1, 158.4, 182.9; UV/Vis (2.6 × 10^−5^ M, CHCl_3_); λ = 405.0 nm (ε, 1.8 × 10^4^).

### 3.16. The DPPH Radical Scavenging Assay

The measurement of the 2,2-Diphenyl-1-picrylhydrazyl (DPPH) radical scavenging effect was performed according to the established procedure [8]. Sample compounds were dissolved in ethanol to obtain a 0.1 mM concentration. The DPPH free radical was dissolved in ethanol to obtain a concentration of 0.2 mM. The ethanol (100 μL) and DPPH solutions (50 μL) were added to a sample solution (100 μL) on a 96-well transparent microplate. The mix solution was mixed on a plate-mixer for 1 min. The mix solution was allowed to stand at 25 °C for 30 min in the dark, followed by measuring the absorbance with a microplate reader at 517 nm. The sample blank test (B) was performed with ethanol instead of the sample solution using a similar procedure. The blank test of the sample (C) was performed similarly, with ethanol instead of the DPPH solution. The blank test of the sample blank (D) was performed similarly, but with ethanol instead of the sample and DPPH solution. The DPPH radical scavenging rate was calculated as follows:

DPPH Radical Scavenging Rate (%) = {(B − D) − (A − C)}/(B − D) × 100(1)

where A is the absorbance of the sample, B is the absorbance of the sample blank, C is the absorbance of the blank of the sample, and D is the absorbance of the blank of the sample blank.

### 3.17. Tyrosinase Activity Inhibition Assay

The Tyrosinase activity was determined using the dopachrome method with l-3-(3,4-dihydroxyphenyl)alanine (l-DOPA) as the substrate [9]. Sample compounds were dissolved in DMSO to obtain a concentration of 3.0 mM. l-DOPA was dissolved in a 0.2 M phosphate buffer solution (PBS, pH 6.8) to obtain a concentration of 1.66 mM. The enzyme tyrosinase from mushrooms was dissolved in PBS to obtain a concentration of 600 units/mL. The sample solution (10 μL) was added to a l-DOPA solution (280 μL) on a 96-well transparent microplate. The mix solution was mixed on a plate-mixer for 1 min. The mix solution was left to stand at 25 °C for 5 min. The tyrosinase solution (10 μL) was added to the mixture and the mixture was incubated at 25 °C for 10 min, followed by measuring the absorbance with a microplate reader at 475 nm. The sample blank test (B) was performed with DMSO instead of the sample solution with similar procedure. The blank test of sample (C) was similarly performed with PBS instead of the enzyme solution. The blank test of the sample blank (D) was similarly performed with the DMSO and PBS instead of the sample and enzyme solutions, respectively. The percentage inhibition of tyrosinase activity was calculated as follows.

Tyrosinase Activity Inhibition Rate (%) = {(B − D) − (A − C)}/(B − D) × 100(2)

where A is the absorbance of the sample, B is the absorbance of the sample blank, C is the absorbance of the blank of the sample, and D is the absorbance of the blank of the sample blank.

## 4. Conclusions

In this study, chalcones, flavanones, and flavonols were easily synthesized, including 8-methoxybutin, which is a naturally occurring product from *Coreopsis lanceolata* L., using the HWE reaction as the key reaction in five to seven steps with overall yields of 18–59%, 13–53%, and 2–21% from *O*-methylpyrogallol **4a**,**b**, respectively. The synthesis of aurones including leptosidin was achieved in four to five steps with overall yields of 5–36% from **4a**,**b** using the aldol condensation reaction as a key reaction.

We found a correlation between the physiological activity and structures of the A- and B-rings of chalcone, flavanone, flavonol, and aurone. Each of chalcones **7b**,**h**; flavanones **8b**,**h**; flavonols **11b**,**h**; and aurones **15b**,**h** with the 3,4-dihydroxy groups on the B-ring had high antioxidant activity. The antioxidant activity in decreasing order was flavonol, chalcone, aurone, and flavanone.

The chalcones **7c**,**i** and aurones **15c**,**i** bearing the 2,4-dihydroxy groups on the B-ring had a high inhibitory activity potential. The whitening effect in decreasing order was chalcone, aurone, flavonol, and flavanone.

## Figures and Tables

**Table 1:** The DPPH radical scavenging assay and tyrosinase inhibition activity assay of chalcones **7a**–**l**.

Entry	Compound	R^1^	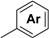	DPPH Radical Scavenging Assay	Tyrosinase Inhibition Activity Assay
				Scavenging Rate (%) ^a^	Inhibition Rate (%) ^b^
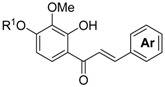					
1	**7a**	H	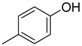	5.8	46.6
2	**7b**	H	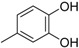	96.0	0.0
3	**7c**	H	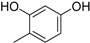	31.6	85.7
4	**7d**	H	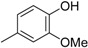	56.7	0.0
5	**7e**	H	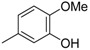	20.5	30.3
6	**7f**	H	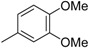	11.4	5.1
7	**7g**	Me	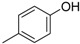	1.0	40.7
8	**7h**	Me	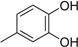	94.0	18.7
9	**7i**	Me	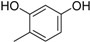	6.9	80.3
10	**7j**	Me	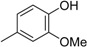	48.8	0.0
11	**7k**	Me	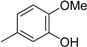	0.1	22.5
12	**7l**	Me	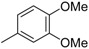	4.2	7.2
13	Lanceolin			94.2 ^c^	
14	α-Tocopherol			95.0	
15	Arbutin				9.3

^a^ Final concentration: 0.040 mM; ^b^ Final concentration: 0.10 mM; ^c^ Tanimoto et al. [1].

**Table 2:** The DPPH radical scavenging assay and tyrosinase inhibition activity assay of flavanones **8a**–**l.**

Entry	Compound	R^1^	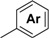	DPPH Radical Scavenging Assay	Tyrosinase Inhibition Activity Assay
				Scavenging Rate (%) ^a^	Inhibition Rate (%) ^b^
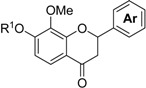					
1	**8a**	H	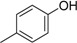	0.0	5.7
2	**8b**	H	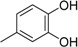	68.4	0.0
3	**8d**	H	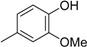	20.3	0.0
4	**8e**	H	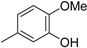	16.2	0.0
5	**8f**	H	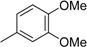	4.7	0.0
6	**8g**	Me	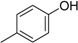	0.6	27.4
7	**8h**	Me	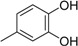	94.2	23.1
8	**8j**	Me	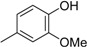	15.6	0.0
9	**8k**	Me	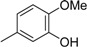	7.2	0.0
10	**8l**	Me	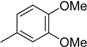	0.0	1.8
11	8-Methoxybutin			94.3 ^c^	
12	α-Tocopherol			95.0	
13	Arbutin				9.3

^a^ Final concentration: 0.040 mM; ^b^ Final concentration: 0.10 mM; ^c^ Okada et al. [2].

**Table 3:** The DPPH radical scavenging assay and tyrosinase inhibition activity assay of flavonols **11a**–**l**.

Entry	Compound	R^1^	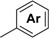	DPPH Radical Scavenging Assay	Tyrosinase Inhibition Activity Assay
				Scavenging Rate (%) ^a^	Inhibition Rate (%) ^b^
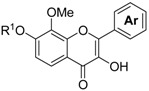					
1	**11a**	H	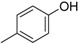	88.1	16.2
2	**11b**	H	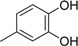	66.5	48.4
3	**11c**	H	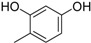	70.3	4.2
4	**11d**	H	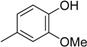	55.0 ^c^	17.1
5	**11e**	H	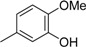	55.3	6.0
6	**11f**	H	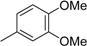	53.6	4.7
7	**11g**	Me	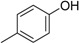	92.5	11.1
8	**11h**	Me	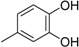	68.6	16.0
9	**11i**	Me	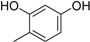	80.6	18.3
10	**11j**	Me	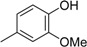	81.5 ^d^	2.7
11	**11k**	Me	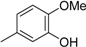	42.1	2.0
12	**10l**	Me	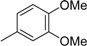	46.4	21.2
13	α-Tocopherol			95.0	
14	Arbutin				9.3

^a^ Final concentration: 0.040 mM; ^b^ Final concentration: 0.10 mM; ^c^ Final concentration: 0.010 mM; ^d^ Final concentration: 0.020 mM.

**Table 4:** The DPPH radical scavenging assay and tyrosinase inhibition activity assay of aurones **14l** and **15a**–**k**.

Entry	Compound	R^1^	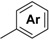	DPPH Radical Scavenging Assay	Tyrosinase Inhibition Activity Assay
				Scavenging Rate (%) ^a^	Inhibition Rate (%) ^b^
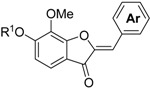					
1	**15a**	H	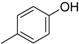	4.8	32.5
2	**15b**	H	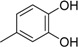	84.2	0.0
3	**15c**	H	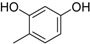	42.8	74.6
4	**15d**	H	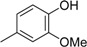	47.4	0.0
5	**15e**	H	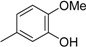	0.0	41.9
6	**15f**	H	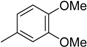	0.0	10.9
7	**15g**	Me	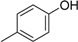	0.2	46.8
8	**15h**	Me	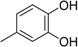	85.8	0.0
9	**15i**	Me	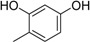	13.3	56.6
10	**15j**	Me	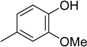	32.8	13.8
11	**15k**	Me	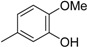	8.9	23.2
12	**14l**	Me	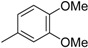	0.5	10.1
13	Leptosidin			93.4 ^c^	
14	α-Tocopherol			95.0	
15	Arbutin				9.3

^a^ Final concentration: 0.040 mM; ^b^ Final concentration: 0.10 mM; ^c^ Okada et al. [2].
